# Effectiveness of digital health interventions on blood pressure control, lifestyle behaviours and adherence to medication in patients with hypertension in low-income and middle-income countries: a systematic review and meta-analysis of randomised controlled trials

**DOI:** 10.1016/j.eclinm.2024.102432

**Published:** 2024-02-01

**Authors:** Vincent Boima, Alfred Doku, Francis Agyekum, Lawrence Sena Tuglo, Charles Agyemang

**Affiliations:** a Department of Medicine and Therapeutics, University of Ghana Medical School, Accra, Ghana; b Department of Public & Occupational Health, University of Amsterdam Medical Centre, University of Amsterdam, Netherlands; c Department of Nutrition and Dietetics, School of Allied Health Sciences, University of Health and Allied Sciences, Ho, Ghana; d Department of Epidemiology, School of Public Health, Nantong University, 9 Seyuan Road, Nantong, Jiangsu, China; e Division of Endocrinology, Diabetes, and Metabolism, Department of Medicine, Johns Hopkins University School of Medicine, Baltimore, MD, USA

**Keywords:** Digital health interventions, Blood pressure control, Lifestyle behaviours, Adherence to medication, Patients with hypertension

## Abstract

**Background:**

Digital health interventions can be effective for blood pressure (BP) control, but a comparison of the effectiveness and application of these types of interventions has not yet been systematically evaluated in low- and middle-income countries (LMICs). This study aimed to compare the effectiveness of digital health interventions according to the World Health Organisation (WHO) classifications of patients in terms of BP control, lifestyle behaviour changes, and adherence to medication in patients with hypertension in LMICs.

**Methods:**

In this systematic review and meta-analysis, we searched the PubMed, Scopus, Web of Science, Embase, CINAHL, and Cochrane Library databases for randomised controlled trials (RCTs) published in English, comprised of adults (≥18 years old) with hypertension and the intervention consisted of digital health interventions according to WHO's classifications for patients in LMICs between January 1, 2009, and July 17, 2023. We excluded RCTs that considered patients with hypertension comorbidities such as diabetes and hypertension-mediated target organ damage (HMTOD). The references were downloaded into Mendeley Desktop and imported into the Rayyan web tool for deduplication and screening. The risk of bias was assessed using Cochrane Risk of Bias 2. Data extraction was done according to Cochrane's guidelines. The main outcome measures were mean systolic blood pressure (SBP) and BP control which were assessed using the random-effect DerSimonian-Laird and Mantel-Haenszel models. We presented the BP outcomes, lifestyle behaviour changes and medication adherence in forest plots as well as summarized them in tables. This study is registered with PROSPERO, CRD42023424227.

**Findings:**

We identified 9322 articles, of which 22 RCTs from 12 countries (n = 12,892 respondents) were included in the systematic review. The quality of the 22 studies was graded as high risk (n = 7), had some concerns (n = 3) and low risk of bias (n = 12). A total of 19 RCTs (n = 12,418 respondents) were included in the meta-analysis. Overall, digital health intervention had significant reductions in SBP [mean difference (MD) = −4.43 mmHg (95% CI −6.19 to −2.67), I^2^ = 92%] and BP control [odds ratio (OR) = 2.20 (95% CI 1.64–2.94), I^2^ = 78%], respectively, compared with usual care. A subgroup analysis revealed that short message service (SMS) interventions had the greatest statistically significant reduction of SBP [MD = −5.75 mm Hg (95% Cl −7.77 to −3.73), I^2^ = 86%] compared to mobile phone calls [MD = 3.08 mm Hg (−6.16 to 12.32), I^2^ = 87%] or smartphone apps interventions [MD = −4.06 mm Hg (−6.56 to −1.55), I^2^ = 79%], but the difference between groups was not statistically significant (p = 0.14). The meta-analysis showed that the interventions had a significant effect in supporting changes in lifestyle behaviours related to a low salt diet [standardised mean difference (SMD) = 1.25; (95% CI 0.64–1.87), I^2^ = 89%], physical activity [SMD = 1.30; (95% CI 0.23–2.37), I^2^ = 94%] and smoking reduction [risk difference (RR) = 0.03; (95% CI 0.01–0.05), I^2^ = 0%] compared to the control group. In addition, improvement in medication adherence was statistically significant and higher in the intervention group than in the control group [SMD = 1.59; (95% CI 0.51–2.67), I^2^ = 97%].

**Interpretation:**

Our findings suggest that digital health interventions may be effective for BP control, changes in lifestyle behaviours, and improvements in medication adherence in LMICs. However, we observed high heterogeneity between included studies, and only two studies from Africa were included. The combination of digital health interventions with clinical management is crucial to achieving optimal clinical effectiveness in BP control, changes in lifestyle behaviours and improvements in medication adherence.

**Funding:**

None.

**Research in context**

**Evidence before this study**

Digital health interventions have shown favourable findings for blood pressure (BP) control. We searched PUBMED, the Cochrane Library, and the PROSPERO database from the start to May 16, 2023, using the search terms (“hypertension” OR “blood pressure” OR “high blood pressure”) AND (“telemedicine” OR “text messaging” OR “electronic health” OR “mobile health” OR mHealth OR “digital health”) AND (“blood pressure control” OR “blood pressure management” OR “hypertension control” OR “hypertension management” OR “medication adherence” OR “smoking cessation” OR “alcohol abstinence” OR “weight loss” OR “diet therapy” OR “behaviour modif∗” OR “physical activit∗”) AND (“randomized controlled trial” OR “controlled trial” OR “trials”) to identify meta-analyses that compared the effectiveness of digital health intervention in adult patients with hypertension, according to World Health Organisation (WHO) classifications, in low- and middle-income countries (LMICs). We found a 2019 meta-analysis of individual global data (n = 4271 respondents) showing that digital health interventions reduced both systolic and diastolic BP in patients with hypertension compared with usual care. That review was limited by the small number of included studies (eleven) and a small pooled sample size (4271), did not compare the effectiveness of digital health interventions according to WHO's classifications for patients and included the majority of the studies from high-income countries. Furthermore, due to rapid innovations in telecommunications, several studies have been published recently in LMICs that have not been included in those reviews and did not compare which type of digital health intervention according to WHO's classifications for patients is effective in LMICs.

**Added value of this study**

To our knowledge, this is the first meta-analysis to compare digital health interventions according to WHO's classifications for patients and report on their effectiveness on BP control, lifestyle behaviour changes, and medication adherence in adult patients with hypertension in LMICs using only randomised controlled trials (RCTs). We found that digital health interventions may be effective in reducing systolic blood pressure (SBP) and BP control in adult patients with hypertension, regardless of the three methods of delivery in LMICs. The meta-analysis also showed that the interventions had a significant effect in supporting changes in lifestyle behaviours related to a low-salt diet, physical activity and smoking reduction as well as improvement in medication adherence compared to the control group. However, we observed high heterogeneity between included studies, and only two studies from Africa were included.

**Implications of all the available evidence**

Since a minimum of 92% of the worldwide population has access to several digital health devices, physicians should familiarise themselves with this method of intervention delivery and encourage patients with hypertension to use scientific digital health devices to improve their BP control. The combination of digital health interventions with clinical management is fundamental to achieving optimal clinical effectiveness in BP control.

## Introduction

Globally, approximately 1.28 billion people are living with hypertension as the second leading risk factor for premature death.^1^^,^^2^ Current guidelines recommend a blood pressure (BP) of 140/90 mmHg or more persistently for the diagnosis of hypertension in adults.^3^ Hypertension is a major cause of cardiovascular disease (CVD), and kidney disease and BP control reduce the risk of these complications.^3^^,^^4^ Despite the availability of effective hypertensive medications, BP treatment and control rates remain low, especially in low- and middle-income countries (LMICs).^4^ Self-management education, including education for patients, self-monitoring of clinical measurements,^5^ lifestyle modifications (e.g., healthy diet, physical activity, weight loss, smoking cessation, and alcohol reduction),^6^ and support for medication adherence, has been extensively used for BP control.^7^

In recent years, digital health interventions have become a very effective, useful and available form of healthcare delivery in self-management and controlling hypertension,^8^, ^9^, ^10^, ^11^, ^12^ compared with usual care. Digital health interventions, also known as “a discrete functionality of digital technology that is applied to achieve health objectives”, have exceptional potential to promote universal health coverage and enhance health service delivery by improving the accountability, availability, accessibility, continuity, utilization, and effectiveness of health care.^13^^,^^14^ The World Health Organisation (WHO) classifies digital health interventions according to types of users (for patients, healthcare providers and data services) to cover various areas of health systems with a particular focus on health service delivery.^15^ One of the WHO's classifications of digital health interventions for patients includes short message service (SMS), multimedia message services (MMS), interactive voice response or phone calls, web-based/online telecare platforms and smartphone applications.^15^ Data show that out of 6.9 billion of the global population, 86% have access to smartphones, 92% use orthodox mobile phones, and 64% have internet access.^16^

One area that has great potential for improvements through digital health interventions is the management of non-communicable diseases (NCDs), including hypertension, in primary health care.^13^ This is because NCDs, particularly hypertension, are characterized by long disease durations and a continuous need to anticipate and alleviate risk factors through lifestyle modifications, which is better addressed by primary health care than higher-level health facilities.^17^ Several meta-analyses reported that digital health interventions reduced both systolic BP and diastolic BP in individuals with hypertension compared with face-to-face delivery.^16^^,^^18^^,^^19^ However, those meta-analyses were inadequate due to the smaller number of studies included from LMICs and the smaller combined sample size. People living in LMICs, such as many countries in Africa, are at high risk of many health conditions compared to those living in high-income countries while having the most limited access to health innovations such as digital health intervention.^20^

Furthermore, due to rapid innovations in telecommunications, several studies have been published recently in LMICs that have not been included in those reviews. Most importantly, although some types of digital health interventions, such as SMS, MMS, interactive voice response or phone calls, web-based/online telecare platforms and smartphone applications, have been used to provide interventions, no study has compared their effectiveness and application to assist reasonable decisions in LMICs. To enable physicians to choose the digital health interventions most effective for BP control, changes in lifestyle behaviours, and improvement in medication adherence, we aimed to compare digital health interventions according to WHO's classifications for patients and report on their effectiveness on BP control, change in lifestyle behaviours, and improvement in medication adherence in adult patients with hypertension in LMICs using only randomised controlled trials (RCTs).

## Methods

### Search strategy and selection criteria

This systematic review and meta-analysis followed and adhered to the appropriate reporting guidelines of the 2020 PRISMA.^21^ The protocol that was followed is registered in PROSPERO, CRD42023424227. The PubMed, Scopus, Web of Science, Embase, CINAHL, and Cochrane Library databases were searched for RCTs published in English using keywords and MeSH terms. The search strategies were maximized to identify articles on patients with hypertension. The searches were limited between January 1, 2009, and July 17, 2023. The reason is that 2009 was designated as the year digital health intervention started to become broadly embraced.^16^ The complete search strategies are presented in the Supplementary File (Table S1 on pages 1–5). We manually searched the reference lists of relevant studies on digital health interventions among patients with hypertension. All references from database searches were downloaded into Mendeley Desktop version 1.19.8. The references were imported from Mendeley into the Rayyan web tool for the removal of duplicates and the remaining articles for eligibility by three authors (LST, AD and VB).

The inclusion criteria were as follows: (1) the population comprised adults (≥18 years old) with hypertension; and (2) the intervention consisted of digital health interventions according to WHO's classifications for patients,^13^ to provide reminders to patients during follow-up to assess BP control, behaviour changes and adherence to medication as well as preventive healthcare services (Table 1). Studies were selected based on interventions focused on primary and secondary outcomes. Interventions must be provided or supported through digital health interventions for patients rather than trials targeting healthcare providers or other stakeholders; (3) the outcome variables include first the intervention aimed at supporting reductions in SBP and BP control; secondarily, changes in lifestyle behaviours (diet, physical activity, weight loss, reduction in body mass index, smoking reduction, alcohol reduction and general quality of life) and improvements in medication adherence; (4) the comparator was usual care, control group or no intervention; (5) the study included data on BP and lifestyle behaviours or medication adherence; and (6) the study design was RCTs. Only digital health interventions implemented among adult patients with hypertension in LMICs according to the World Bank ranking and published in English were considered for inclusion.^39^ We excluded RCTs that considered patients with hypertension comorbidities such as diabetes and hypertension-mediated target organ damage (HMTOD), such as strokes, hypertensive heart disease, ischaemic heart diseases, retinopathy and chronic kidney disease. Nevertheless, the findings of this review can be applied to patients with hypertension with comorbidities, as confirmed by previous meta-analyses conducted among hypertensive patients with other chronic conditions.^40^, ^41^, ^42^, ^43^ Additionally, studies that did not use digital health interventions according to the WHO's classifications for patients were excluded.^13^

**Table 1:** Characteristics of the included study interventions and outcomes.

Reference; country; sample size at follow up	Study design; duration; mHealth type	Study intervention	Outcomes		Conclusion
			Primary	Secondary	
Bobrow et al., 2016^8^; South Africa; CG: 396 IG1: 406 IG2: 394	Single-blind RCT; 12 months; SMS	Two intervention groups (informational only and interactive). Informational only received SMS to encourage adherence to medication; interactive received all informational-only messages; control (no text)	Δ SBP −1.1 mm Hg (control), −3 mm Hg (informational only), −2.9 mm Hg (interactive); informational only vs. control, p = 0.05; interactive vs. control, p = 0.16; proportion BP control for informational only vs. control (OR = 1.42; p = 0.03) and for interactive vs. control (OR = 1.41; p = 0.04)	Satisfaction with treatment for informational only vs. control was (OR 0; p = 0.99) and interactive vs. control was (OR 0; p = 0.99); medication changes for informational only vs. control was (OR 1.2; p = 0.26) and interactive vs. control was (OR 1.04; p = 0.78)	mHealth effective for BP control
Jahan et al., 2020^9^; Bangladesh; CG: 208 IG: 204	Prospective single-centre RCT; 5 months; SMS	SMS for education and behaviour changes motivation (e.g., PA for 30 min at least 5 days a week, healthier diet, and medication adherence); SMS reminders for behaviour changes based on the DASH diet	Δ SBP −11.2 mm Hg (intervention) and −8.6 mm Hg (control; p = 0.04); Δ DBP −5.0 mm Hg and −4.4 mm Hg (control; p = 0.02)	Adherence rate of salt intake was 67% (intervention) and 76% (control; p = 0.04) and PA was 73% (intervention) and 82% (control; p = 0.03)	mHeatlh effective for BP control
Rehman et al., 2019^22^; Pakistan; CG: 60 IG:60	Prospective RCT; 3 months; SMS	SMS text for lifestyle modifications, on nutrition education, PA, and motivation; daily SMS reminder to take medicine on time; weekly SMS requesting BP report	Δ SBP −8 mm Hg (intervention) and −2 mm Hg (control); Δ DBP −6 mm Hg (intervention) and −3 mm Hg (control); no statistical significance reported	The intervention group reported feeling “fresher and more energetic” and having a “better mood” at the end of the study vs. the control group	Could not be assessed with reported data
Gong et al., 2020^10^; China; CG: 218 IG: 225	Multicenter RCT; 6 months; Smartphone app	A smartphone app that provides drug dose and BP measurement reminders	Δ SBP −9.0 mm Hg (intervention) and −5.9 mm Hg (control; p = 0.05); Δ DBP −7.0 mm Hg (intervention) and −4.1 mm Hg (control; p = 0.05); Percentage of participants with controlled BP 77% (intervention) and 67% (control; p = 0.01)	Medication adherence was 55% (low), 42% (medium), and 3% (high) in intervention and 68% (low), 30% (medium), and 2% (high) in control (p < 0.0001)	mHealth effective for BP control
Li et al., 2019^11^; China; CG: 143 IG: 110	Prospective cluster RCT; 6 months; Smartphone app	WeChat lasting >1 h; health education; health promotion; group chat; BP monitoring	Δ SBP −5.3 mm Hg (intervention) and −1.6 mm Hg (control; p < 0.0001); Δ DBP −1.1 mm Hg (intervention) and 2.0 mm Hg (control; p = 0.02); BP control 84% (intervention) and 64% (control; p < 0.0001); BP monitoring (≥1/week) 78% (intervention) and 57% (control; p < 0.0001)	Hypertension knowledge (intervention vs. control AMD 1.5, p = 0.11); self-efficacy (intervention vs. control AMD 1.4, p = 0.09); self-management (intervention vs. control AMD 8.7, p < 0.0001); Social support (intervention vs. control AMD −0.3, p = 0.31)	mHealth effective for BP control
Sun et al., 2020^23^; China; CG: 60 IG: 60	RCT, 3 months; Smartphone app	Patients stratified into three WeChat groups according to cardiovascular risk (low, middle, and high); health education; health behaviour promotion; group chats; BP monitoring	Δ SBP −11 mm Hg (intervention) and −3.4 mm Hg (control; p < 0.0001); Δ DBP −5.7 mm Hg (intervention) and −2.2 mm Hg (control; p = 0.02)	Δ BMI −0.5 kg/m^2^ (intervention; p < 0.0001) and −0.1 kg/m^2^ (control; p = 0.05); Δ LDL-C = 0.1 mmol/L (intervention; p = 0.02) and 0.01 mmol/L (control; p = 0.47)	mHealth effective for BP control
Wang et al., 2020^24^; China; CG: 75 IG: 76	Multicenter RCT; 6 months; SMS	Automated SMS to improve patients' health behaviours; health belief education	Δ SBP −10.8 mm Hg (intervention) and −1.3 mm Hg (control; p = 0.01); Δ DBP −0.8 mm Hg (intervention) and −0.3 mm Hg (control; p = 0.01)	A total score of health behaviour for intervention (3.2 ± 0.4) and control (2.5 ± 0.5), p < 0.0001. Medication adherence for intervention (3.9 ± 0.4) and control (3.0 ± 1.0), p = 0.02	mHealth effective for BP control
Wan et al., 2018^25^; China; CG: 78 IG: 80	Multicenter RCT; 3 months; SMS	Multiple reminding methods to improve patients' health behaviour/health belief levels and informs the patient of his/her current health conditions	Δ SBP −9.9 mm Hg (intervention) and −1.4 mm Hg (control; p = 0.03); Δ DBP −0.6 mm Hg (intervention) and 3.1 mm Hg (control; p = 0.07); BP Control 80% (intervention) and 46% (control; p < 0.0001).	PA for intervention (2.6 ± 0.6) and control (2.1 ± 0.7), p < 0.0001. Low-salt diet for intervention (3.5 ± 0.7) and control (3.1 ± 1.0), p < 0.0001. Medication adherence for intervention (3.9 ± 0.3) and control (3.5 ± 0.7), p < 0.0001.	mHealth effective for BP control
Bhandari et al., 2022^12^; Nepal; CG: 75 IG: 79	Unblinded RCT; 3 months; SMS	Patients received SMS text information on hypertension, physical activity, a diet low in salt and reminders to take medication	Δ SBP −8.0 mm Hg (intervention) and −1.0 mm Hg (control; p < 0.0001); Δ DBP −5.8 mm Hg (intervention) and −1.7 mm Hg (control; p < 0.0001); BPs control 70% (intervention) and 48% (control; p = 0.01).	Improvement in compliance to antihypertensive therapy (p < 0.0001), medication adherence (p < 0.0001), medication adherence self-efficacy (p = 0.02) and knowledge of hypertension and its treatment (p = 0.01)	mHealth effective for BP control
Kingue et al., 2013^26^; Cameroon; CG: 103 IG: 165	Prospective RCT; 6 months; Mobile phone call	Mobile telephone communication on nonpharmacological interventions such as weight reduction in overweight and obese patients, dietary sodium reduction, smoking cessation, alcohol reduction and increasing physical activity	SBP 169.2 ± 27.9 for (intervention) and 160.8 ± 23.7 (control; p = 0.01); DBP 100.4 ± 18.3 for (intervention) and 95.2 ± 14.8 (control; p = 0.01)	Waist circumference 92.1 ± 16.0 for (intervention) and 93.0 ± 19.4 (control; p = 0.66); BMI kg/m^2^ 27.3 ± 6.4 (intervention) and 29.4 ± 12.6 (control; p = 0.09)	mHealth effective for BP control
Piette et al., 2012^27^; Honduras and Mexico; CG: 92 IG: 89	RCT; 1.5 months; Mobile phone call	Automated call reminders to check BP regularly; medication adherence; and intake of salty foods.	Δ SBP −10.6 mm Hg (intervention) and −6.4 mm Hg (control; p = 0.09); Δ DBP −7.3 mm Hg (intervention) and −4.1 mm Hg (control; p = 0.08); BP Control 57% (intervention) and 38% (control; p = 0.01).	Satisfaction with hypertension care (OR 2.9; p < 0.0001); BP medication adherence was 89% (intervention) and 77% (control; p = 0.04).	mHealth effective for BP control
Pan et al., 2018^28^; China; CG: 55 IG: 52	RCT; 6 months; Smartphone app	Smartphone application installed to receive home telemonitoring for BP	Δ SBP −16.4 mm Hg (intervention) and −9.8 mm Hg (control); Δ DBP −7.4 mm Hg (intervention) and −4.4 mm Hg (control); BP Control 64% (intervention) and 42% (control; p = 0.03).	The adherence rate to antihypertensive medications was 49% (intervention) and 12% (control)	mHealth effective for BP control
Zhai et al., 2020^29^; China; CG: 192 IG: 192	Cluster RCT; 3 months; SMS	Personal consultations by trained pharmacy students; SMS every 3 days; intervention group and control group were given standard pharmaceutical care according to the Guidelines for Good Pharmacy Practice	Δ SBP −11.5 mm Hg (intervention) and −9.2 mm Hg (control; p < 0.0001); Δ DBP −0.3 mm Hg (intervention) and −2.7 mm Hg (control; p = 0.06); BP Control 56% (intervention) and 44% (control; p = 0.03).	8-item MMAS score for intervention (7.4 ± 1.2) and control (7.0 ± 1.3, p = 0.04); knowledge score 0.44 (p < 0.0001); Satisfaction with BP control change 70%	mHealth effective for BP control
Yuting et al., 2023^30^; China; CG: 68 IG: 66	RCT; 3 months; Smartphone app	Smartphone application to receive information about hypertension and health promotion, a home-based BP monitor wearable wristband that stored and uploaded BP data to a secure website via Bluetooth.	Δ SBP −8.5 mm Hg (intervention) and −1.3 mm Hg (control); Δ DBP −0.4 mm Hg (intervention) and −0.01 mm Hg (control)	Δ WC (intervention vs. control AMD −1.8, p < 0.0001); Δ HC (intervention vs. control AMD −0.3, p = 0.08); hypertension compliance (intervention vs. control AMD 3.9, p < 0.0001); self-efficacy (intervention vs. control AMD 7.9, p < 0.0001); physical health, (intervention vs. control AMD 10.5, p < 0.0001); mental health, (intervention vs. control AMD 10.9, p < 0.0001)	mHealth effective for BP control
He et al., 2017^31^; Argentina; CG: 648 IG: 709	Cluster RCT; 18 months; SMS	Community health worker-led home-based intervention (health coaching and home BP monitoring and audit), physician education and BP feedback, and individualized weekly SMS to promote lifestyle changes and reinforce medication adherence	Δ SBP −19.3 mm Hg (intervention) and −12.7 mm Hg (control; p < 0.0001); Δ DBP −12.2 mm Hg (intervention) −6.9 mm Hg (control; p < 0.0001)	The proportion of controlled hypertension was 73% (intervention) and 52% (control); adherence to antihypertensive medication was 66% (intervention) and 53% (control)	mHealth effective for BP control
Maslakpak et al., 2016^32^; Iran; CG: 41 IG1: 41 IG2: 41	RCT; 3 months; SMS	Two intervention groups (SMS and reminder cards) provide a relationship with patients and remind them to take their medication; control (no intervention)	Data on BP not reported	Adherence to treatment for SMS (57.7 ± 2.8), and reminder cards (57.5 ± 2.7) vs. control (46.6 ± 3.0) (p < 0.0001). Tracking drug regimen for SMS (5.6 ± 1.0), reminder card (5.7 ± 1.0) vs. control (5.5 ± 0.9) (p < 0.0001). Follow-up medical appointment for SMS (6.7 ± 0.9), reminder cards (6.7 ± 0.8) vs. control (5.5 ± 0.9) (p < 0.0001). Follow the diet for SMS (11.7 ± 0.5); reminder cards (11.5 ± 1.0) vs. control (10.0 ± 1.1) (p < 0.0001).	Improved adherence to medication and diet
Bozorgi et al., 2021^33^; Iran; CG: 60 IG: 58	RCT; 6 months; Smartphone app	Smartphone application for recording BP and receiving feedback on the recorded BP, healthy diet (DASH) and weight loss plans	Data on BP not reported	Adherence to medication/self-assessment for intervention (19.7 ± 0.7) and control (18.0 ± 1.5); Adherence to a low-salt diet for intervention (18.6 ± 0.9) and control (17.1 ± 1.4). BMI (kg/m^2^) for intervention (28.6 ± 3.2) and control (28.4 ± 3.7). PA for intervention (247.3 ± 96.3) and control (102.7 ± 53.3)	Improved adherence to medication and diet
Zhang et al., 2022^34^; China; CG: 88 IG: 104	RCT; 6 months; Smartphone app	Bracelet with an installed smartphone application for recording BP, tracing data on DASH diet and checking weight.	SBP 19% for (intervention) and 14% (control); DBP 26.0% for (intervention) and 14% (control)	Adherence to weight reduction for intervention (66.7 ± 11.0) control (64.1 ± 12.4); Adherence to BMI management for intervention (25.5 ± 3.4) and control (25.6 ± 3.8);	mHealth effective for BP control
David et al., 2023^35^; Brazil; CG: 42 IG: 174	RCT; 6 months; SMS	SMS text instructions for the DASH diet of low salt intake, physical activity, alcohol reduction, smoking cessation and telemonitoring of BP.	Data on BP not reported	Adherence to weight for intervention (81.7 ± 17.2) and control (87.6 ± 21.5). Adherence to smoking cessation for intervention was 87% and control was 83%. Adherence to moderate or no alcohol intake (RR 1.1, p = 0.04). The adherence rate of low salt intake was 22% (intervention) and 17% (control). The adherence rate of PA was 67% (intervention) and 43% (control).	Improved adherence to physical activity, no alcohol intake, diet quality
Kes et al., 2021^36^; Turkey; CG: 38 IG: 39	Single-blind RCT; 3 months; SMS	A personalized SMS test was sent to remind respondents of their prescribed medication	SBP 69% for (intervention) and 21% (control, p < 0.0001); DBP 82% for (intervention) and 32% (control, p < 0.0001)	Adherence to medication for intervention (41.9 ± 5.6) and control (28.6 ± 6.0, p < 0.0001); Adherence to DASH diet (p = 0.29); Adherence to smoking for intervention 28% and control (21%, p = 0.47). Adherence to alcohol consumption (p = 0.18); Adherence to PA (p = 0.19)	mHealth effective for BP control
Zhou et al., 2022^37^; China; CG: 1133 IG: 2985	Cluster RCT; 12 months; SMS	SMS was sent to remaining patients about adherence to medicine therapy, lifestyle changes and BP control	The intervention effect −10.1 mm Hg for SBP and −1.8 mm Hg. The rate of BP control rate is 47% (intervention) and 30% (control)	Increase the use of antihypertensive medications (OR 1.06, p = 0.02). Adherence to smoking cessation (OR 0.96, p = 0.33); Adherence to alcohol reduction (OR 0.99, p = 0.76); Adherence to PA (OR 1.00, p = 0.86)	mHealth effective for BP control
Ionov et al., 2020^38^; Russia; CG: 80 IG: 160	Prospective RCT; 3 months; Smartphone app	Mobile applications installed on the smartphone were given to patients to measure their BP while patients in the control group continued with usual care.	AMD Δ SBP −16.8 ± 2.9 and 7.9 ± 3.9 mm Hg, (p < 0.0001); SBP Δ −7.8 ± 3.0 and 3.6 ± 4.2 mm Hg, (p < 0.0001); BP control for 69% (intervention) and 25% (control)	Increase the health-related quality of life (p = 0.04); Improved patient-perceived quality of care (p < 0.0001).	mHealth effective for BP control

CG: control group; IG: intervention group; RCT: randomized control trial; SMS: short message service; Δ: change; SBP: systolic blood pressure; DBP: diastolic blood pressure; BP: blood pressure; OR: odds ratio; RR: relative risk; DASH: dietary approaches to stop hypertension; AMD: adjusted mean difference; 8-item MMAS score: 8-item Morisky Medication Adherence Scale score; PA: physical activity; BMI: body mass index; LDL-C: low-density lipoprotein-cholesterol.

### Data analysis

Three authors (LST, AD and VB) developed a comprehensive data extraction form according to the guidelines led in the Cochrane Handbook.^44^ We extracted the data, such as authors' names, publication year, study details (country, design, masking and randomization method, retention rate, and statistical analyses), participants' characteristics (condition, inclusion and exclusion criteria, sample size, recruitment process, and demographics (age and gender)), intervention (type, duration, and main and secondary result), comparison (description of care), outcomes (primary and secondary outcomes [with means and SDs, proportion and total], and decisions of the effectiveness of the digital health intervention. Outcome data were extracted for measurements of SBP, DBP and BP control, as well as lifestyle behaviours and other changes in physical measurements (diet, physical activity, weight loss, reduction in body mass index, smoking cessation, alcohol reduction) and medication adherence (Table 1 and Supplementary File, Table S3 on pages 17–18). Age was grouped for subgroup analysis in this review based on the WHO's report that an estimated 1.28 billion adults aged 30–79 years (average 54 years) worldwide have hypertension, the majority of whom (two-thirds) live in LMICs,^2^ in the Supplementary File, Table S4, on pages 22–23).

The relevant study characteristics, results, intervention and reports on the effectiveness of the intervention were summarized. The intervention was categorised as effective if the digital health intervention had statistically significant (p ≤ 0.05) effects compared to usual care and considered ineffective if no significant differences were found between the main outcomes (p ≥ 0.05). The main outcome was mean SBP (and 95% Cl) and BP control. The secondary outcomes were changes in lifestyle behaviours (diet, physical activity, weight loss, body mass index, smoking reduction, alcohol reduction and general quality of life) and improvement in medication adherence.

The quality of the studies was assessed by three authors (LST, AD and VB) using the reviewed Cochrane risk-of-bias 2.^45^ These are selection bias (random sequence generation and allocation concealment); performance bias (blinding of respondents and personnel); detection bias (blinding of outcome assessment); attrition bias (incomplete outcome data); reporting bias (selective reporting); and bias that emerges from period and carryover effects (for crossover studies).^45^ Any disagreements were resolved by the fourth author FA (Supplementary File, Figs. S1 and S2, on pages 18 and 19).

STATA Version 17, R Version 4.3.2 and Review Manager Version 5.4.1 were used for the analyses. The I^2^ statistic and Cochran's Q test were used to assess heterogeneity. Depending on the heterogeneity of the data, random-effect (for I^2^ ≥ 50%) or fixed-effect (for I^2^ < 50%) models were used. Effect sizes for continuous outcomes were calculated using the mean difference (MD) for SBP and DBP and the standardised mean difference (SMD) and risk difference (RR) for lifestyle behavioural and medication adherence outcome measurements. Effect sizes for dichotomous outcome measurements were calculated using the odds ratio (OR) for BP control, lifestyle behaviour and medication adherence. Subgroup analyses by different modes of digital health intervention, duration of digital health intervention, year of publication and income economies were performed to determine potential sources of heterogeneity. Sensitivity analyses were performed to determine the strength of the pooled estimates and whether a single study was responsible for the outcomes. Funnel plots were used to assess publication biases visually and statistically by Egger's and Begg's tests for confirmation at p ≤ 0.05.

### Role of the funding source

There was no funding source for this study. VB, AD and FA had access to the dataset and had final responsibility for the decision to submit it for publication.

## Results

**Fig. 1:**
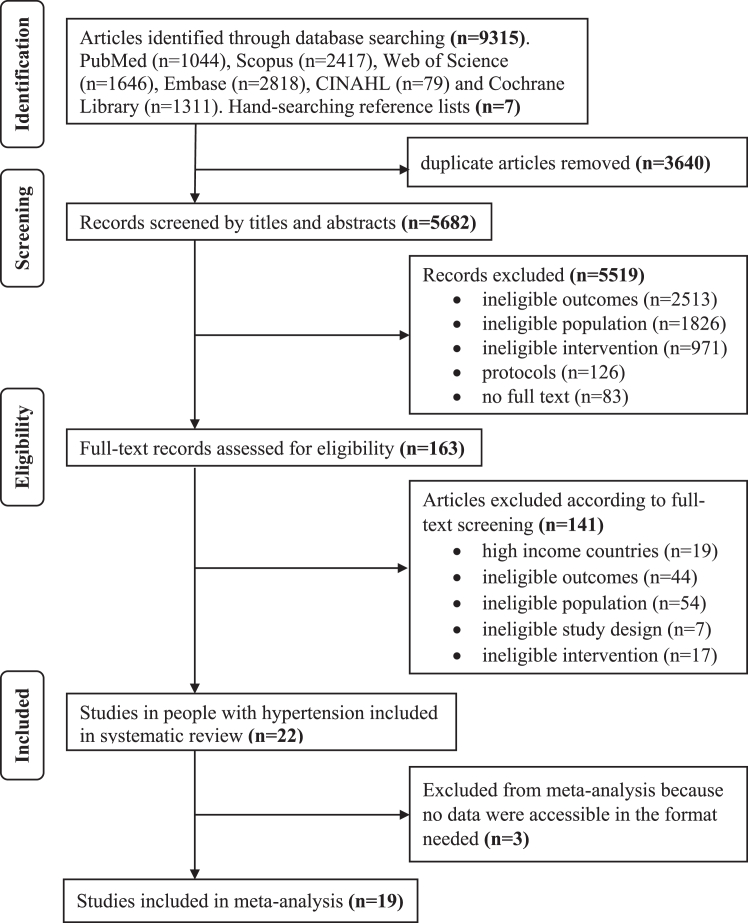
PRISMA flow chart of study selection. PRISMA flow diagram specifying the considerations to exclude and include the articles.

The database searches returned 9315 articles, and an additional 7 articles were found through manual searching of reference lists of pertinent articles. We removed 3640 duplicates, 5682 titles and abstracts were screened, 5519 records were excluded together with articles without full texts, 163 full texts were assessed for eligibility, and 141 that focused on people with other health conditions and other reasons listed were excluded. Finally, 22 studies on adult patients with hypertension were captured in the systematic review (Fig. 1).

### Characteristics of the included studies

From the 22 studies, 12,892 participants from 12 countries were included and published from 2012 to 2023.^8^, ^9^, ^10^, ^11^, ^12^^,^^22^, ^23^, ^24^, ^25^, ^26^, ^27^, ^28^, ^29^, ^30^, ^31^, ^32^, ^33^, ^34^, ^35^, ^36^, ^37^, ^38^ Most studies (n = 10) were conducted in China,^10^^,^^11^^,^^23^, ^24^, ^25^^,^^28^, ^29^, ^30^^,^^34^^,^^37^ and (n = 2) in Iran.^32^^,^^33^ One study each was conducted in South Africa,^8^ Bangladesh,^9^ Pakistan,^22^ Nepal,^12^ Cameroon,^26^ Honduras and Mexico,^27^ Brazil,^35^ Turkey,^36^ Russia,^38^ and Argentina.^31^ Fifteen studies were conducted in upper-middle-income economies (UMIEs),^8^^,^^10^^,^^11^^,^^23^, ^24^, ^25^^,^^28^, ^29^, ^30^, ^31^^,^^34^, ^35^, ^36^, ^37^, ^38^ and 7 in lower-middle-income economies (LMIEs),^9^^,^^12^^,^^22^^,^^26^^,^^27^^,^^32^^,^^33^ according to the World Bank classification.^39^ Only RCT articles were considered, with 14 employing a matching design, 3 a cluster, and 5 prospective. A total of 12 studies adopted SMS as the main method for the provision of digital health intervention,^8^^,^^9^^,^^12^^,^^22^^,^^24^^,^^25^^,^^29^^,^^31^^,^^32^^,^^35^, ^36^, ^37^ 8 used smartphone apps (apps installed on smartphones),^10^^,^^11^^,^^23^^,^^28^^,^^30^^,^^33^^,^^34^^,^^38^ and 2 used mobile phone calls.^26^^,^^27^ The period of the intervention varied from 1.5 to 18 months, less than 12 months in 19 studies (83.3%),^9^, ^10^, ^11^, ^12^^,^^22^, ^23^, ^24^, ^25^, ^26^, ^27^, ^28^, ^29^, ^30^^,^^32^, ^33^, ^34^, ^35^, ^36^^,^^38^ and 12 months and more in 3 studies (16.7%),^8^^,^^31^^,^^37^ (Table 1 and Supplementary File, Table S3 on pages 17–18).

### Assessment of bias of the articles included

Out of 22 articles included, 7, three and 12 were categorised as high risk, having some concerns, and low risk of bias, respectively. Three articles were considered to have a high risk of bias for the randomization procedure.^8^^,^^11^^,^^32^ One study assigned respondents before allocation concealment,^32^ and two provided partial information about random sequence generation.^8^^,^^11^ Four deviated from the proposed intervention, without blinding the respondents and personnel to the intervention task,^11^^,^^22^^,^^24^^,^^28^; however, such blinding is usually not feasible in digital health interventions. Two articles did not report on the blinding of respondents.^22^^,^^24^ Five articles reported high attrition biases due to missing data.^10^^,^^11^^,^^23^^,^^28^ Four articles acquired a high risk of bias categorisation for adopting a selective reporting of results, lack of information on sufficient training of personnel for the assessment of results, or not stating if results evaluators were mindful of respondents’ intervention task.^23^^,^^24^^,^^26^ Regarding the choice of reported outcomes, two articles provided incomplete result assessments of data analysis, causing a high risk of bias.^11^^,^^26^ Generally, the majority of the SMS intervention articles were measured as low risk, but most of the smartphone apps and mobile phone call intervention articles were evaluated as high risk (Supplementary File, Figs. S1 and S2, on pages 18 and 19).

### Meta-analysis

Nineteen studies (n = 12,418 participants) were included in the meta-analysis,^8^, ^9^, ^10^, ^11^, ^12^^,^^22^, ^23^, ^24^, ^25^, ^26^, ^27^, ^28^, ^29^, ^30^, ^31^^,^^34^^,^^36^, ^37^, ^38^ and 5 out of 19 were measured as having a high risk of bias.^8^^,^^11^^,^^22^^,^^24^^,^^28^ Two studies reported two interventions against the same outcome assessed.^8^^,^^32^

### Blood pressure

**Fig. 2:**
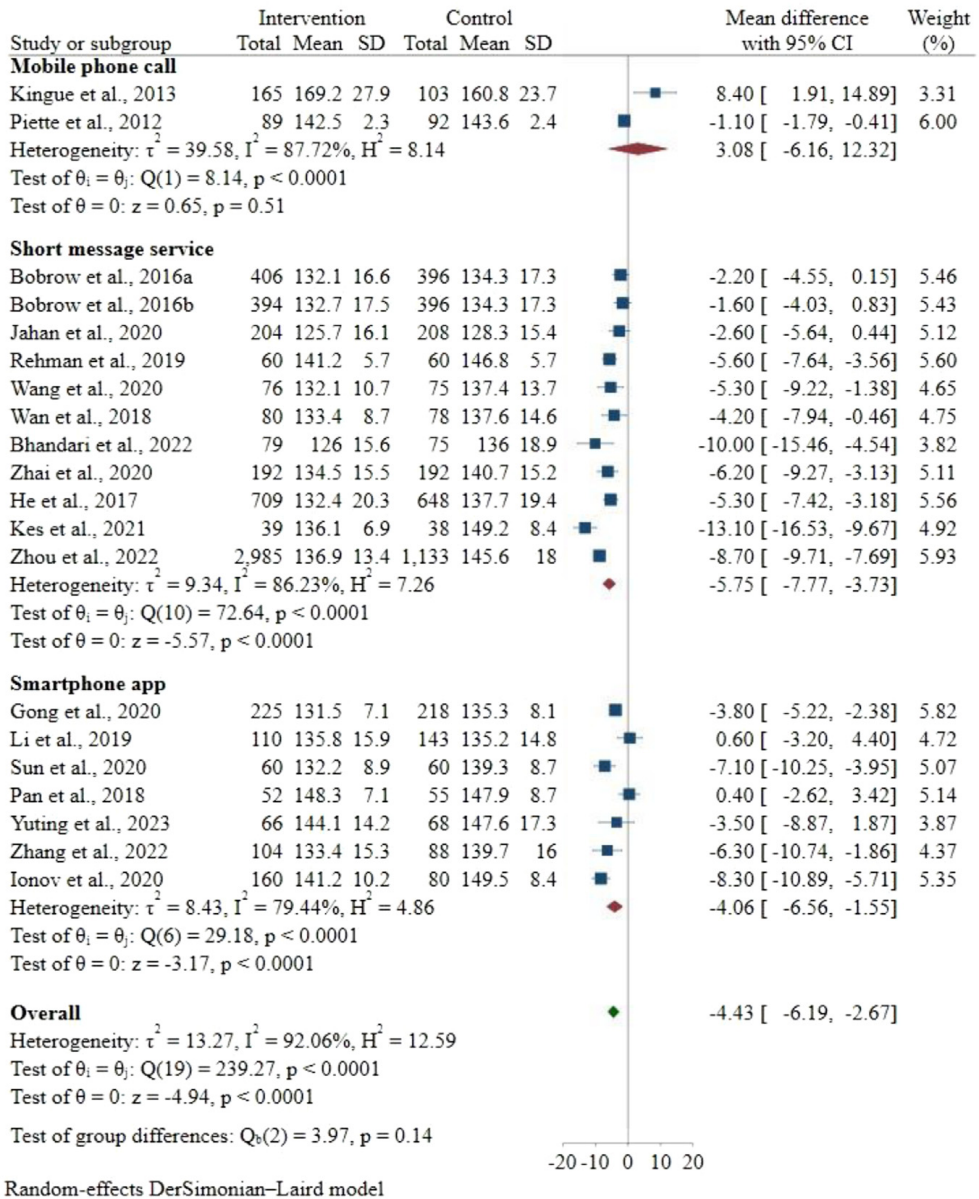
Forest plot of the mean difference in SBP between the intervention and control groups. Forest plot of mean difference in systolic blood pressure (SBP) (expressed as mm Hg) between the digital health intervention and the control groups, and subgroup analysis by mode of delivery of the intervention (Mobile phone call, Short message service (SMS) and Smartphone app). The size of the blue squares indicates the weight of the evidence from each of the studies. Studies with CI (horizontal line) crossing zero (vertical line) are inconclusive. Studies with more participants have narrower CIs. The red diamonds represent the summary effect sizes in each of the subgroups and the green the overall sample, with the width of the diamond indicating the 95% CI. A statistically significant greater reduction in SBP is seen in the intervention group, compared with the control group in the overall sample and with the two modes of delivery (SMS and smartphone app). SMS interventions displayed the greatest reduction, compared with smartphone apps and mobile phone calls, but the differences between the three modes were not significant. The data present high heterogeneity.

**Fig. 3:**
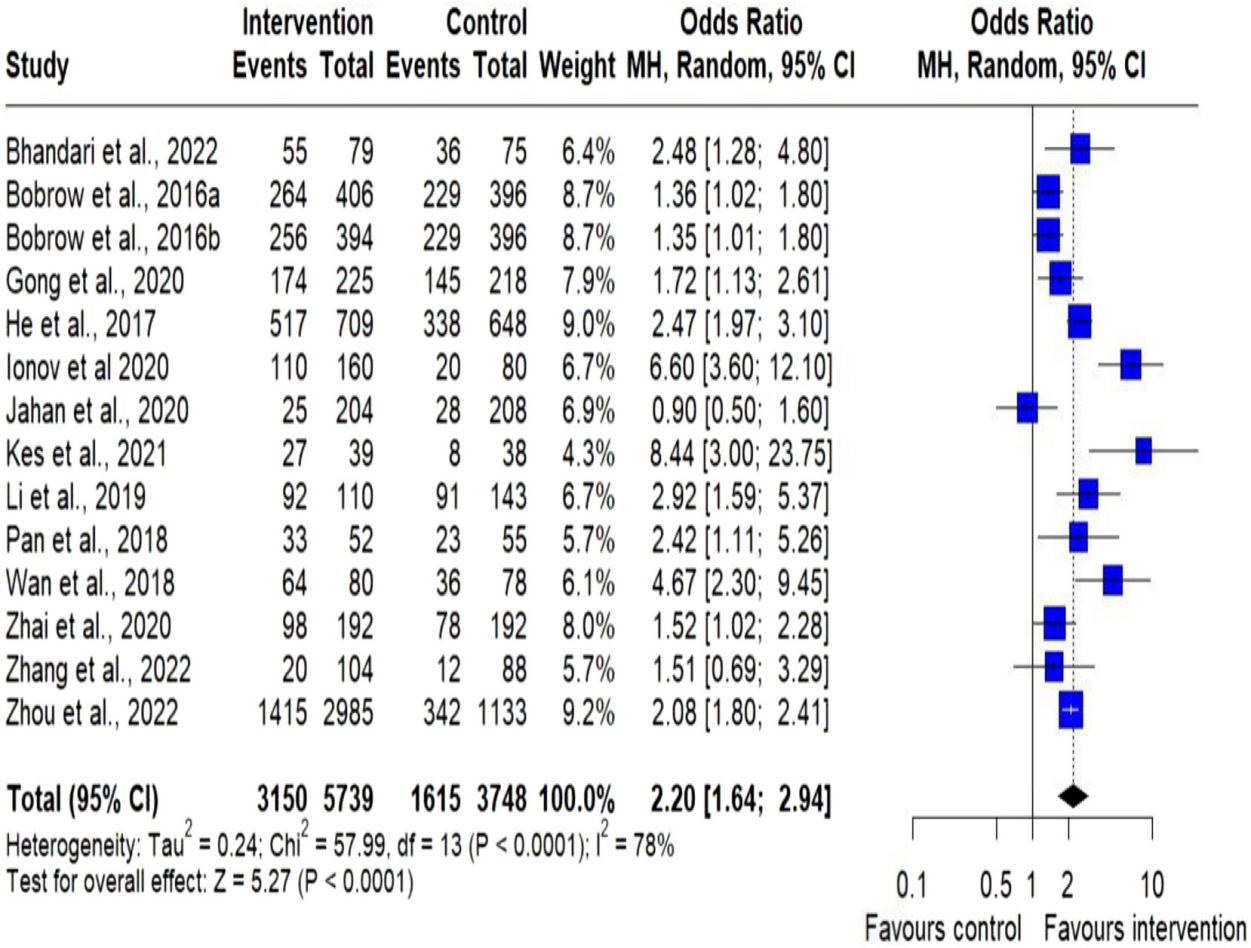
Meta-analysis of dichotomous outcome measurements for BP control. Forest plot of odds ratio in blood pressure (BP) control between the digital health intervention and the control groups. The size of the blue squares indicates the weight of the evidence from each of the studies. Studies with CI (horizontal line) crossing zero (vertical line) are inconclusive. Studies with more participants have narrower CIs. The black diamonds represent the summary effect sizes in the overall sample, with the width of the diamond indicating the 95% CI. A statistically significant greater reduction in BP is seen in the intervention group, compared with the control group. The data present high heterogeneity.

The study by,^8^ reported two interventions against the same outcome assessed and included in the meta-analysis of SBP. Therefore, 20 interventions were reported on the forest plot of SBP,^8^, ^9^, ^10^, ^11^, ^12^^,^^22^, ^23^, ^24^, ^25^, ^26^, ^27^, ^28^, ^29^, ^30^, ^31^ 17 interventions for DBP,^9^, ^10^, ^11^, ^12^^,^^22^, ^23^, ^24^, ^25^, ^26^^,^^28^, ^29^, ^30^, ^31^ and 14 interventions for the comparison of BP control.^8^, ^9^, ^10^, ^11^, ^12^^,^^25^^,^^27^, ^28^, ^29^^,^^31^^,^^34^^,^^36^, ^37^, ^38^ Overall, the digital health intervention showed a [mean difference (MD) = −4.43 mm Hg (95% CI −6.19 to −2.67); n = 10,461] significant reduction in SBP compared with the control. This variance in reduction between the intervention and usual care was not statistically significant in the combined effect (mobile phone call, SMS, and smartphone app) or each of the two individual methods of providing digital health interventions (SMS and smartphone app). Subgroup analysis showed that interventions that used SMS revealed better SBP reduction (MD = −5.75 mm Hg [−7.77 to −3.73]; n = 8496) than interventions that employed mobile phone calls (MD = 3.08 mm Hg [–6.16 to 12.32]; n = 449) or smartphone apps (MD = −4.06 mm Hg [−6.56 to −1.55]; n = 1489), but the difference between groups was not statistically significant (p = 0.14). The pooled heterogeneity across the studies was significant (Q = 239.3; p < 0.0001) and high (I^2^ = 92.1%). Mobile phone call studies (I^2^ = 87.7%) and SMS studies (I^2^ = 86.2%) showed larger heterogeneity than smartphone app studies (I^2^ = 79.4%) (Fig. 2). Overall, participants receiving digital health intervention achieved a significant reduction in DBP [MD = −2.06 mm Hg (95% CI −3.37 to −0.75); n = 8688] compared with the control. The reduction difference between the interventions and usual care was statistically significant in the combined effect (mobile phone calls, SMS and smartphone app studies) and each of the three individual methods of providing mHealth interventions. SMS interventions showed the highest DBP reduction compared with the control (MD = −3.02 mm Hg [−4.25 to −1.79]; n = 6931), followed by smartphone apps (MD = −1.15 mm Hg [−4.09 to 1.78]; n = 1489) and mobile phone calls (MD = 5.20 mm Hg [1.01–9.39]; n = 268). The variation across groups was significant (p < 0.0001). The pooled heterogeneity across the groups was statistically significant (Q = 156.3; p < 0.0001) and high (I^2^ = 89.8%). SMS studies (I^2^ = 79.7%) displayed greater heterogeneity than smartphone app studies (I^2^ = 93.1%). The mobile phone call intervention did not provide any statistically significant results compared with usual care (Supplementary File, Fig. S3, on page 20). The odds of having BP control in the intervention group [odds ratio (OR) = 2.20; (95% CI 1.64–2.94); p < 0.0001; n = 9487] compared with the control group. Significantly high heterogeneity was shown (X^2^ = 58.0; p < 0.0001; I^2^ = 78.0%) (Fig. 3).

### Lifestyle behaviours

#### Low salt diets

**Fig. 4:**
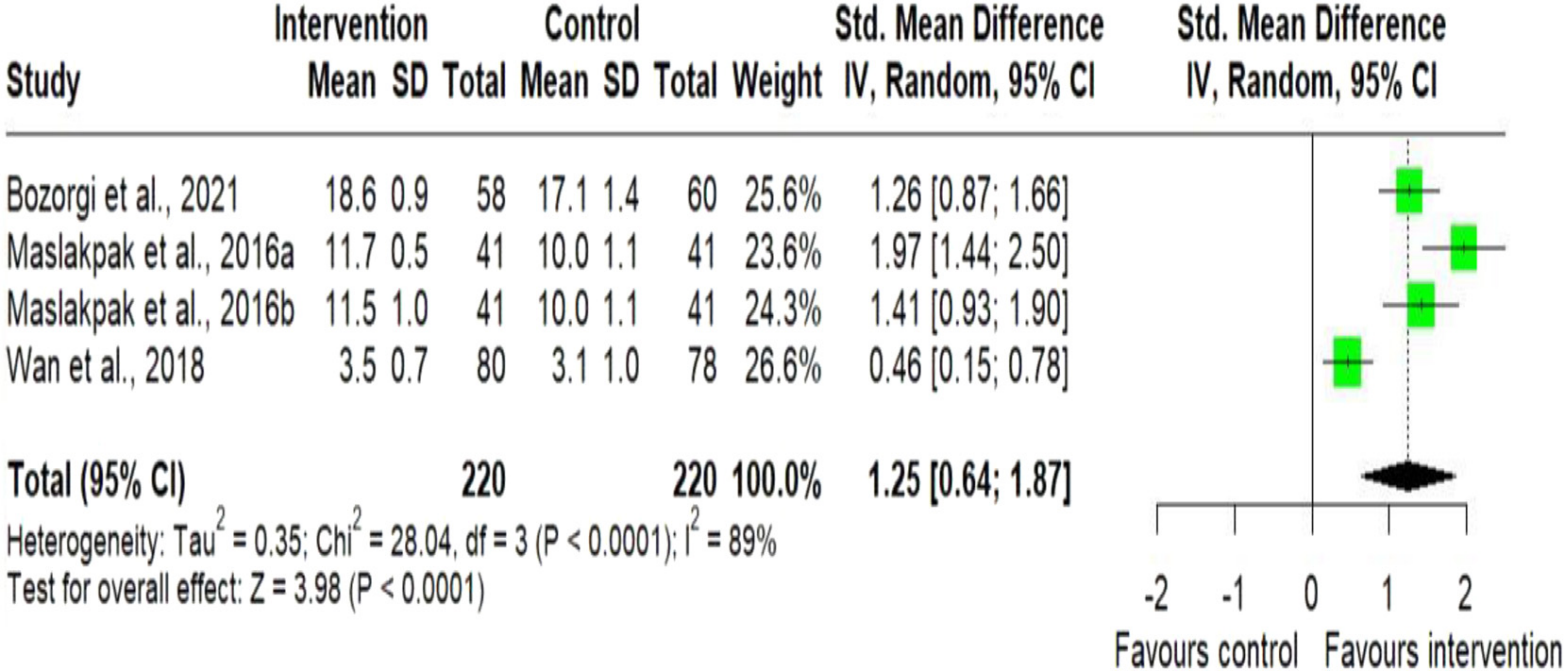
Meta-analysis of continuous outcome measurements for low-salt diets. Forest plot of standard mean difference for low-salt diets between the digital health intervention and the control groups. The size of the green squares indicates the weight of the evidence from each of the studies. Studies with CI (horizontal line) are inconclusive. The black diamonds represent the summary effect sizes in the overall sample, with the width of the diamond indicating the 95% CI. A statistically significant greater reduction for low-salt diets is seen in the intervention group, compared with the control group. The data present high heterogeneity.

A total of 6 studies out of 22,^9^^,^^25^^,^^32^^,^^33^^,^^35^^,^^36^ reported the impact of digital health intervention on healthy diets (low salt diets). The meta-analysis of continuous outcome measurements for low salt diets of 3 studies showed significant differences in the intervention [SMD = 1.25; (95% CI 0.64–1.87); p < 0.0001; n = 440] compared to the control group,^9^^,^^35^^,^^36^ (Fig. 4). However, the meta-analysis of 3 studies that used dichotomous outcome measurements for low salt intake showed no significant differences in the intervention [OR = 0.71; (95% CI 0.49–1.04); p = 0.08; n = 705] compared to the control group,^25^^,^^32^^,^^33^ (Supplementary File, Fig. S4, on page 21).

#### Physical activity

**Fig. 5:**
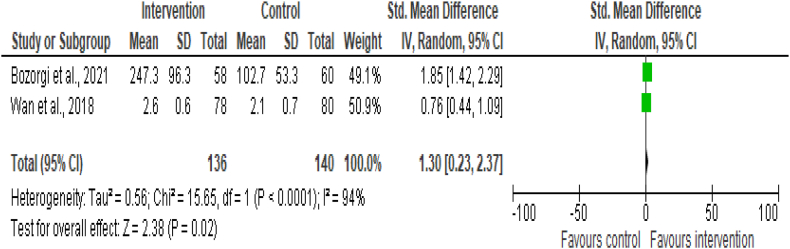
Meta-analysis of continuous outcome measurements for PA. Forest plot of standard mean difference for physical activity (PA) between the digital health intervention and the control groups. The size of the green squares indicates the weight of the evidence from each of the studies. Studies with CI (horizontal line) crossing zero (vertical line) are inconclusive. The black diamonds represent the summary effect sizes in the overall sample, with the width of the diamond indicating the 95% CI. A statistically significant greater reduction for PA is seen in the intervention group, compared with the control group. The data present high heterogeneity.

A total of 6 studies out of 22,^9^^,^^25^^,^^33^^,^^35^, ^36^, ^37^ measured the effectiveness of digital health intervention on physical activity (PA). The meta-analysis of continuous outcome measurements for PA in 2 studies showed significant differences in the intervention [SMD = 1.30; (95% CI 0.23–2.37); p = 0.02; n = 276] compared to the control group,^25^^,^^33^ (Fig. 5). However, the meta-analysis of 4 studies that used dichotomous outcome measurements for PA showed no significant differences in the intervention [OR = 1.53; (95% CI 0.75–3.12); p = 0.24; n = 4823] compared to the control group,^9^^,^^35^, ^36^, ^37^ (Supplementary File, Fig. S5, on page 21).

#### Weight loss

A total of 2 studies out of 22 evaluated the effectiveness of digital health interventions on weight loss. The meta-analysis of continuous outcome measurements for weight loss of 2 studies showed no significant differences in the intervention [SMD = −0.04; (95% CI −0.58 to 0.49); p = 0.87; n = 408] compared to the control group,^34^^,^^35^ (Supplementary File, Fig. S6, on page 21).

#### Reduction in body mass index

A total of 4 studies out of 22 assessed the effectiveness of digital health interventions on body mass index (BMI). The meta-analysis of continuous outcome measurements for BMI of 4 studies showed no significant differences in the intervention [SMD = −0.13; (95% CI −0.28 to 0.02); p = 0.09; n = 698] compared to the control group,^23^^,^^26^^,^^33^^,^^34^ (Supplementary File, Fig. S7, on page 22).

#### Smoking reduction

**Fig. 6:**
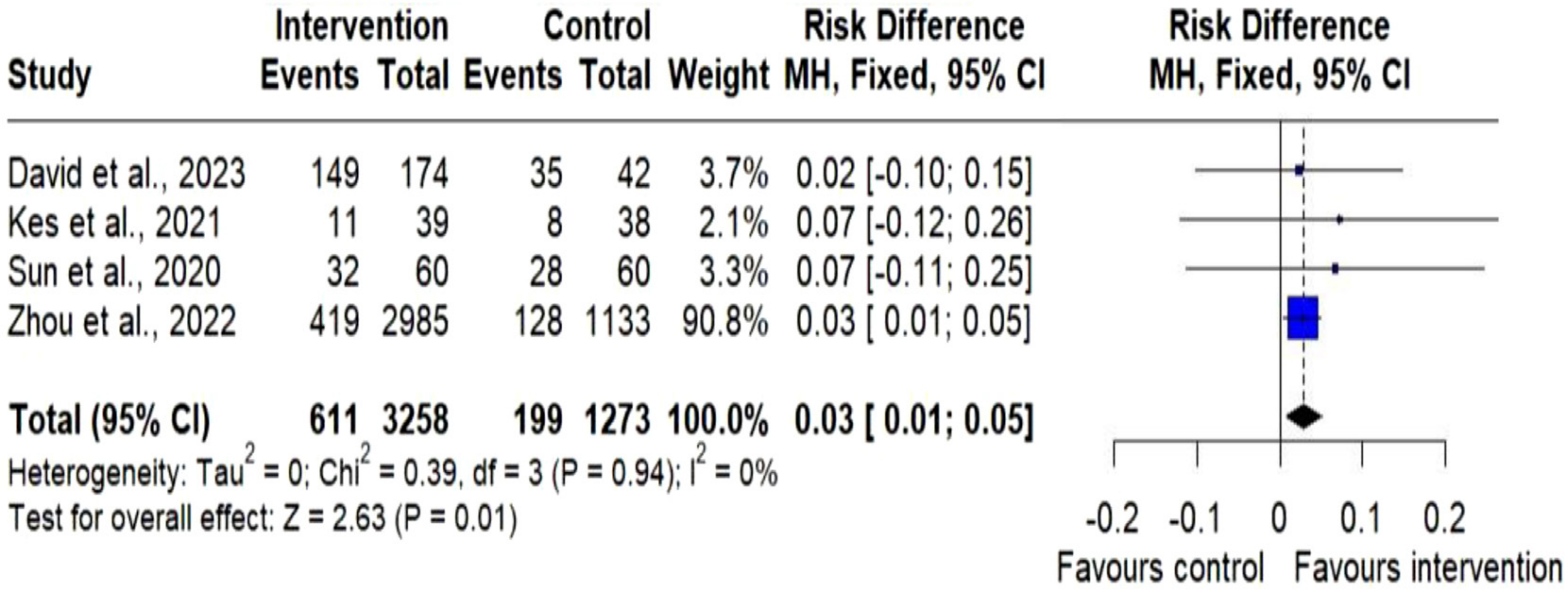
Meta-analysis of dichotomous outcome measurements for smoking reduction. Forest plot of risk difference for smoking reduction between the digital health intervention and the control groups. The size of the blue squares indicates the weight of the evidence from each of the studies. Studies with CI (horizontal line) crossing zero (vertical line) are inconclusive. The black diamonds represent the summary effect sizes in the overall sample, with the width of the diamond indicating the 95% CI. A statistically significant greater reduction in smoking reduction is seen in the intervention group, compared with the control group. The data present no heterogeneity.

A total of 4 studies out of 22 assessed the effectiveness of digital health interventions on smoking reduction. The meta-analysis of dichotomous outcome measurements for smoking reduction in 4 studies revealed significant differences in the intervention [RR = 0.03; (95% CI 0.01–0.05); p = 0.01; n = 4531] compared to the control group,^23^^,^^35^, ^36^, ^37^ (Fig. 6).

#### Alcohol reduction

A total of 3 studies out of 22 assessed the effectiveness of digital health interventions on alcohol reduction. The meta-analysis of dichotomous outcome measurements for alcohol reduction in 3 studies revealed no significant differences in the intervention [RR = 0.01; (95% CI −0.01 to 0.04); p = 0.26; n = 4411] compared to the control group,^35^, ^36^, ^37^ (Supplementary File, Fig. S8, on page 22).

#### Adherence to medication

**Fig. 7:**
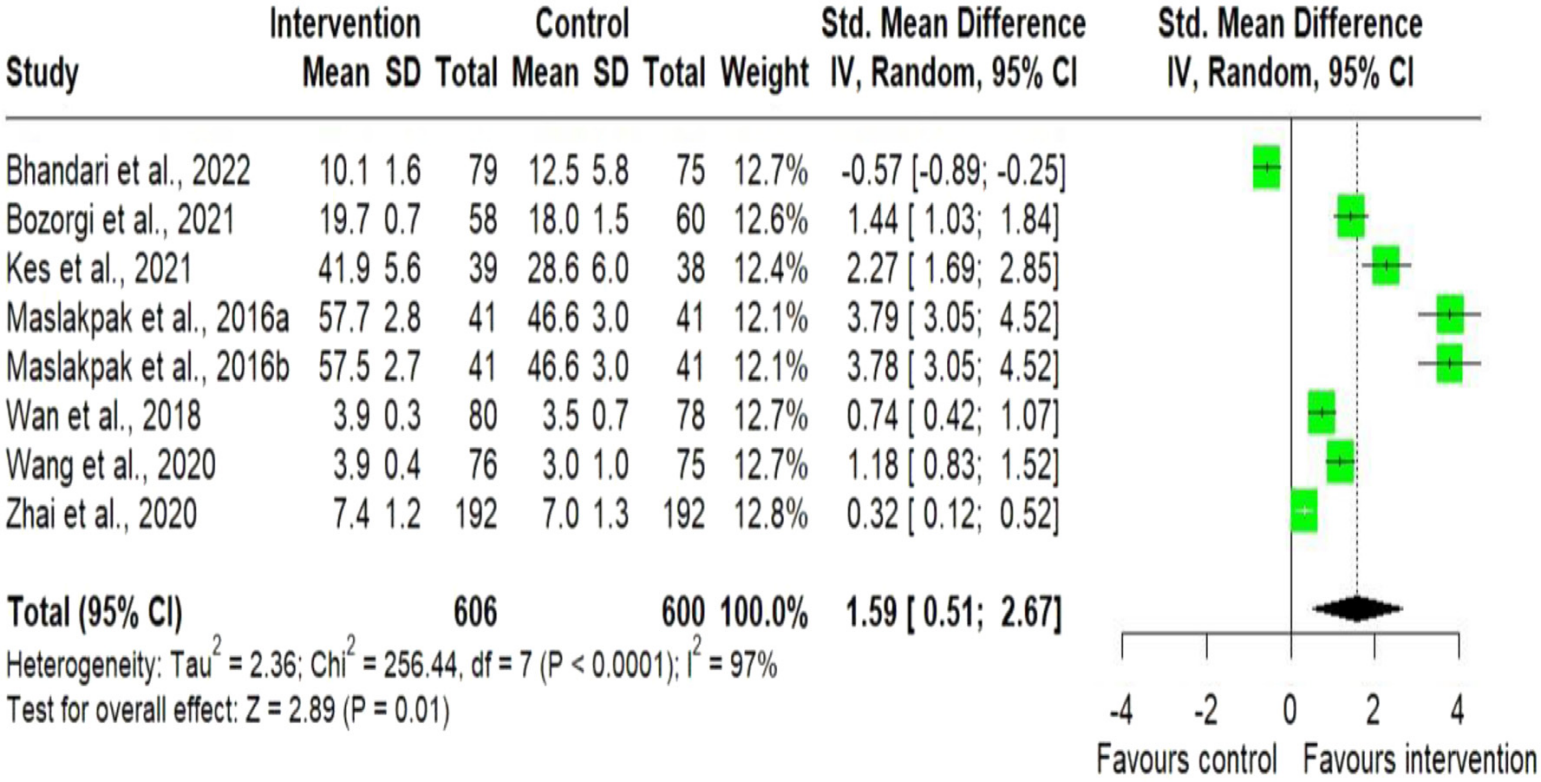
Meta-analysis of continuous outcome measurements for adherence to medication. Forest plot of standard mean difference for continuous outcome measurements for adherence to medication between the digital health intervention and the control groups. The size of the green squares indicates the weight of the evidence from each of the studies. Studies with CI (horizontal line) crossing zero (vertical line) are inconclusive. The black diamonds represent the summary effect sizes in the overall sample, with the width of the diamond indicating the 95% CI. A statistically significant greater reduction in adherence to medication is seen in the intervention group, compared with the control group. The data present high heterogeneity.

**Fig. 8:**
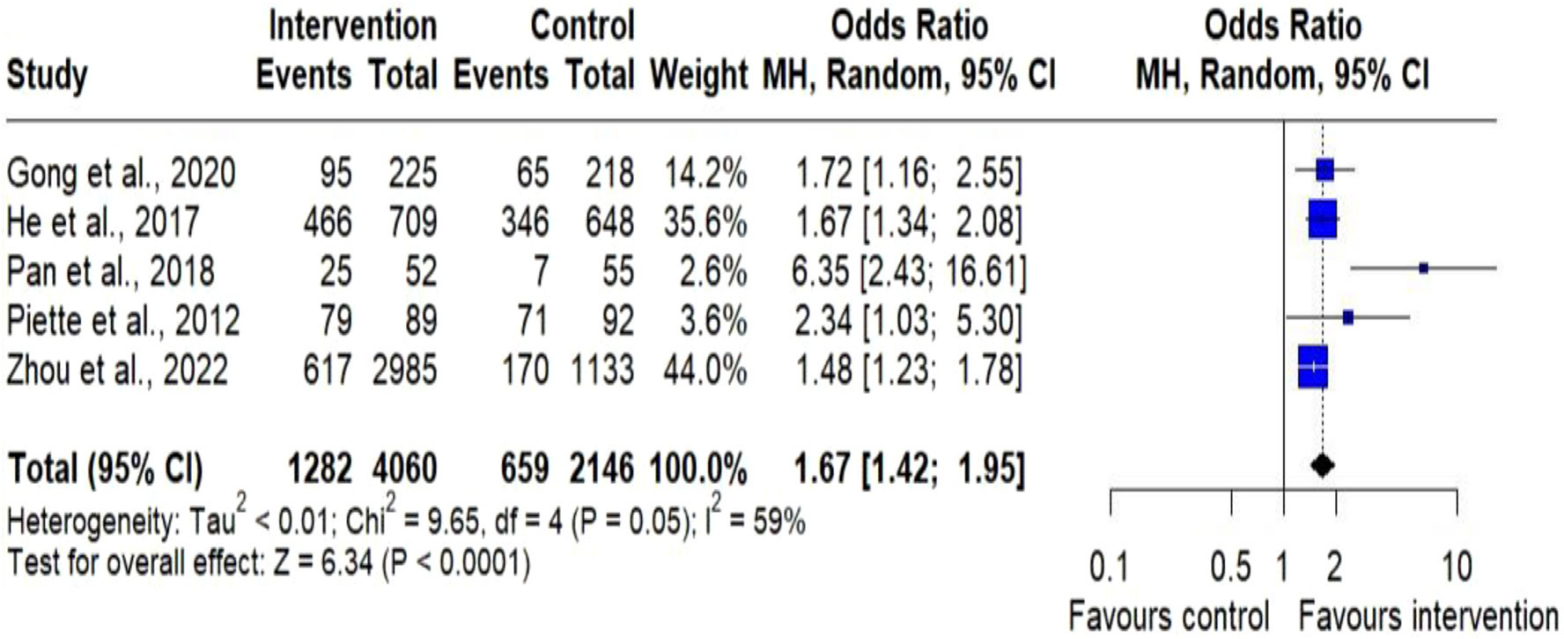
Meta-analysis of dichotomous outcome measurements for adherence to medication. Forest plot of standard mean difference for dichotomous outcome measurements for adherence to medication between the digital health intervention and the control groups. The size of the blue squares indicates the weight of the evidence from each of the studies. Studies with CI (horizontal line) are inconclusive. The black diamonds represent the summary effect sizes in the overall sample, with the width of the diamond indicating the 95% CI. A statistically significant greater reduction in adherence to medication is seen in the intervention group, compared with the control group. The data present moderate heterogeneity.

A total of 12 studies,^10^^,^^12^^,^^24^^,^^25^^,^^27^, ^28^, ^29^^,^^31^, ^32^, ^33^^,^^36^^,^^37^ out of 22 were found to assess BP as an outcome measure along with medication adherence using different measurement scales (Table 1). Two studies,^24^^,^^25^ measured adherence to medication using the Health Promoting Lifestyle Profile II (HPLP II) Chinese version, originally developed by Walker and Hill-Polerecky,^46^ three studies,^12^^,^^32^^,^^33^ used the Hill Bone compliance scale,^47^ one study,^36^ used the Medication Adherence Self–Efficacy Scale Short Form (MASES-SF) developed by,^48^ four studies,^10^^,^^27^^,^^29^^,^^31^ used the 8-item Morisky Medication Adherence Scale,^49^ and two studies,^28^^,^^37^ used a standardized questionnaire. The meta-analysis of continuous outcome measurements for medication adherence was greater in the intervention group [SMD = 1.59; (95% CI 0.51–2.67); p < 0.0001; n = 1206] than in the usual care group,^12^^,^^24^^,^^25^^,^^29^^,^^32^^,^^33^^,^^36^ (Fig. 7). A similar direction of effect was found for studies that used dichotomous outcome measurements for medication adherence, suggesting 1.67 times more likely to achieve clinical medication adherence [OR = 1.67; (95% CI 1.42–1.95); p < 0.0001; n = 6206] compared to the control group,^10^^,^^27^^,^^28^^,^^31^^,^^37^ (Fig. 8).

#### Subgroup analyses

Subgroup analyses by income economies, according to the World Bank ranking,^39^ duration of digital health intervention, and publication year were performed to assess possible causes of heterogeneity. Compared with usual care, all interventions showed significantly better reductions across the studies in SBP and DBP for all groups analysed. Differences between the subgroups were not statistically significant except for SBP for publication year and country for DBP. There was high heterogeneity across the studies, which cannot be explained. The overall SBP (−5.01 mm Hg [−6.83 to −3.20]) and DBP (−2.15 mm Hg [−3.70 to −0.60]) reductions were greater in studies conducted in UMIEs,^8^^,^^10^^,^^11^^,^^23^, ^24^, ^25^^,^^28^, ^29^, ^30^, ^31^^,^^34^^,^^36^, ^37^, ^38^ than in studies conducted in LMIEs.^9^^,^^12^^,^^22^^,^^26^^,^^27^ Studies with interventions that lasted 12 months and beyond,^8^^,^^31^^,^^37^ showed greater reductions in both SBP (−4.55 mm Hg [−8.29 to −0.81]) and DBP (−3.11 mm Hg [−5.06 to −1.16]) than studies that lasted below 12 months.^9^, ^10^, ^11^, ^12^^,^^22^, ^23^, ^24^, ^25^, ^26^, ^27^, ^28^, ^29^, ^30^^,^^34^^,^^36^^,^^38^ RCT studies published after 2019,^9^^,^^10^^,^^12^^,^^23^^,^^24^^,^^29^^,^^30^^,^^34^^,^^36^, ^37^, ^38^ revealed a greater pooled effect of both SBP (−6.79 mm Hg [−8.68 to −4.91]) and DBP (−2.77 mm Hg [−4.45 to −1.09]) than trials published in 2019 and before.^8^^,^^11^^,^^22^^,^^25^, ^26^, ^27^, ^28^^,^^31^ Studies with a proportion of males greater than 50% and above,^10^^,^^12^^,^^24^^,^^25^^,^^30^^,^^38^ showed greater reductions in both SBP (−5.65 mm Hg [−7.78 to −3.51]) and DBP (−3.38 mm Hg [−5.89 to −0.87]) than studies with a proportion of males of less than 50%.^8^^,^^9^^,^^11^^,^^23^^,^^26^, ^27^, ^28^, ^29^^,^^31^^,^^34^^,^^36^^,^^37^ These studies have more male representatives as study participants than females. Studies with a population aged below 54 years,^9^^,^^12^^,^^23^^,^^36^ showed greater reductions in both SBP (−8.07 mm Hg [−12.85 to −3.30]) and DBP (−4.37 mm Hg [−7.06 to −1.67]) than studies with a population aged above 60 years.^11^^,^^29^^,^^30^^,^^37^ RCT studies conducted in China,^10^^,^^11^^,^^23^, ^24^, ^25^^,^^28^, ^29^, ^30^^,^^34^^,^^37^ showed greater reductions in SBP (−4.52 mm Hg [−6.77 to −2.26]) compared to studies conducted in other countries,^8^^,^^9^^,^^12^^,^^22^^,^^26^^,^^27^^,^^31^^,^^36^^,^^38^ (Supplementary File, Table S4, on pages 22–23).

#### Sensitivity analyses

Sensitivity analyses to determine the strength of the pooled effect for the SBP and DBP results were performed. We sequentially removed each study to assess whether the direction of associations was influenced by a single large study with a positive outcome. For the SBP outcome, the direction of associations did not change with the removal of any of the study datasets, with the calculated effect sizes ranging from −3.99 [−5.71 to −2.27] to −4.69 [−6.49 to −2.89] mm Hg reductions in SBP. Overall, the effect interventions (combined phone call, SMS and smartphone app) estimate was not sensitive to individual studies. No individual study affected the direction of associations or the statistical significance of the effect within each subgroup. We also tested whether the fixed-effects model would yield different results from the random-effects model. The fixed-effects model produced a greater reduction in SBP for the overall summary effect with a narrower confidence interval (−3.88 [−4.31 to −3.45] mm Hg) (Supplementary File, Fig. S4, on page 24). For the DBP outcome, the direction of associations did not change with the removal of any of the study datasets, with the calculated effect sizes ranging from −1.75 [−3.08 to −0.42] to −2.38 [−3.67 to −1.10] mm Hg reductions in DBP. Overall, the effect (combined phone call, SMS and smartphone application interventions) estimate was not sensitive to individual studies. No individual study affected the direction of associations or the statistical significance of the effect within each subgroup. We also tested whether the fixed-effects model would yield different results from the random-effects model. The fixed-effects model produced a greater reduction in DBP for the overall summary effect with a narrower confidence interval (−3.06 [−3.43 to −2.69] mm Hg) (Supplementary File, Fig. S5, on page 25).

#### Publication bias

Publication biases were determined visually by funnel plots. The forest plots for both SBP and DBP showed slight asymmetry and more symmetric dispersal, respectively (Figs. 3 and 6). These results were confirmed statistically by Egger's and Begg's tests. For SBP, Egger's test (p = 0.47) and Begg's test (p = 0.92) and for DBP, Egger's test (p = 0.39) and Begg's test (p = 0.59). Therefore, either the SBP or the DBP funnel plot specified publication bias (Supplementary File, Figs. S6 and S7, on pages 26 and 27).

## Discussion

This study presents the comparative effectiveness and application of digital health interventions according to the WHO's classifications for patients and reports on their effectiveness in BP control, changes in lifestyle behaviours, and improvements in medication adherence in adult patients with hypertension in LMICs. The meta-analysis of 19 studies (n = 10,461 respondents) resulted in better BP control, with a significant reduction in SBP and DBP by 4.43 mm Hg and 2.06 mm Hg, respectively, compared with the control. The decreases in SBP and DBP were scientifically significant. First, it confirmed that digital health was effective in controlling and managing hypertension, medication adherence, healthy lifestyles and body composition.^50^^,^^51^ According to the Blood Pressure Lowering Treatment Trialists' Collaboration analysis of 29 RCTs, a reduction in SBP and DBP of 2 mmHg would remarkably decrease the prevalence of CVD by 10%.^18^ Our findings are consistent with previous meta-analyses performed among patients with hypertension. A study of 12 RCTs showed that digital health intervention produced greater reductions in SBP by 3.96 and DBP by 1.85 mm Hg than the control.^19^ A meta-analysis of 11 RCTs revealed that digital health intervention resulted in a remarkable decrease in SBP by 3.85 mmHg and in DBP by 2.19 mmHg compared to usual care.^52^ Similarly, a study with global individual data (n = 7092 respondents) confirmed that digital health intervention provides a notable reduction in SBP by 3.62 and DBP by 2.45 mm Hg compared with the control.^16^ The similarity across the studies could be attributed to the inclusion of some respondents on antihypertensive drugs.

When comparing the three various methods of delivery, we found that SMS and smartphone apps were more effective in terms of BP control than phone call interventions due to the small number of phone call RCT studies. However, SMS presented the highest reduction in SBP and DBP compared with smartphone app interventions. In contrast, a study by Siopis et al.,^16^ comparing the efficacy of SMS, smartphone app, and website interventions on improving BP in adult patients with hypertension found that smartphone app and website interventions provided a greater remarkable reduction in SBP and DBP compared with SMS interventions. The disparities between these studies are that our study was centred on LMICs, whereby more people cannot afford smartphones with applications, compared with the study by Siopis et al.,^16^ which assessed digital health interventions globally. Second, due to challenges in accessing internet services, more people in LMICs own a telephone with SMS capacity compared to smartphones.^16^ In LMICs, cell phone penetration has surpassed 90% in recent years, and mobile internet connectivity is approximately 40%.^53^ Third, SMS might be more user-friendly and easier to access due to the convenient and portable nature of mobile phones, especially in LMICs, compared to smartphone apps or websites that might require knowledge to operate and access networks. Even those with smartphones find it difficult to use the applications frequently because of the high cost of data. However, the reduction in SBP and DBP due to smartphone application in our systematic review might reflect patients’ preferences and their affordability of data and internet services. Additionally, the satisfactoriness of SMS and smartphone app interventions by patients with hypertension has been established in healthcare delivery.^12^^,^^54^

We found the odds of BP control to be 2.20 times greater in the delivery of digital health intervention compared to usual care. Similarly, a study by Li et al.,^19^ found the odds of BP control to be 1.42 times higher in the delivery of digital health intervention compared to usual care. Kassavou et al.,^55^ also found significant and higher odds of 1.60 times BP control in the intervention compared to the control group. These similarities could be due to the presence of respondents with controlled BP. In addition, the significance of BP control applying digital health intervention could also be due to the BP values at baseline in the studies included, which showed that those with inadequate BP control might also benefit from digital health intervention. Our findings provide evidence that digital health intervention could be an important strategy for BP control in LMICs. We found greater reductions both in SBP and DBP for studies conducted in UMIEs than in studies conducted in LMIEs. The finding affirmed a study by Mourtzinis et al.,^56^ that patients with hypertension in the lowest income quantile had a lower likelihood of achieving the BP target than those in the highest quantile. The reason may be that lower income was associated with a reduced likelihood of achieving BP control,^56^ as a large proportion (31%) of households in low-income countries were unable to afford two BP-lowering medicines compared to 9% in middle-income countries and 1% in high-income countries.^57^

This systematic review shows that trials with interventions that lasted 12 months and beyond resulted in greater reductions in both SBP and DBP compared to studies that lasted less than 12 months. Our findings disagree with a study by Li et al.,^19^ who reported greater SBP reduction in studies that lasted less than 12 months. However, a study by Lu et al.,^52^ found no statistically significant reductions in SBP in studies with interventions that lasted less than 12 months. This implies that digital health interventions should be started as early as possible and sustained for a longer period. Although it is believed that 2009 was considered when digital health interventions began and were widely accepted,^16^ of our subgroup meta-analyses showed that studies published after 2019 of digital health applications showed greater reductions in BP control compared to studies published before 2019. This could be attributed to the advancement in technology, which is altering the means healthcare services are provided, from wearable devices (BP monitors smartwatches, etc.) that deliver earlier diagnoses and treatment.

The wide accessibility and affordability of using mobile devices with SMS capacity reported in this review show the possible impact of a digital health intervention on lifestyle changes to reduce hypertension-related morbidity and mortality in LMICs. Studies have shown that digital health interventions result in changes in lifestyle behaviours as an effective tool for improving adherence,^55^^,^^58^^,^^59^ which is consistent with the present review. In our meta-analysis, we found that digital health interventions showed significant effects for improvements in a healthy diet by reducing high salt intake. This finding concurs with the study by Kassavou et al.,^55^ who found positive effects for supporting improvements in a healthy diet by reducing the consumption of high-sodium food. Physical inactivity is independently associated with 12% of the global burden of hypertension.^51^ Thus, physical activity is measured as the basis on which changes in lifestyle to prevent cardiovascular disease must be based. Therefore, the finding in this meta-analysis is promising because the use of digital health interventions is seen to result in favourable changes in lifestyle behaviour such as physical activity, which can have a positive impact on the secondary prevention of future cardiovascular events.^60^ This finding is inconsistent with a study by Kassavou et al.,^55^ that found a moderate but insignificant effect of app-based behavioural self-monitoring interventions in improving physical activity. Our meta-analysis for smoking reduction revealed significant differences in the intervention compared to the control group. This finding agreed with the study by Kassavou et al.,^55^ that showed improvement in smoking cessation among those receiving an app-based self-monitoring intervention compared to those in the control group. Regarding the study by Kassavou et al.,^55^ only one study was found to report the percentage as significantly higher in the intervention group compared to the usual care group.

The effectiveness of digital health interventions on adherence to medication was reported in previous systematic reviews with an improvement in medication adherence and other health outcomes.^59^^,^^61^^,^^62^ Our meta-analysis showed that the digital health intervention of medication adherence increased the odds of achieving medication adherence twofold in the intervention group compared to the control group. Our finding is similar to the finding by Kassavou et al.,^55^ who found a significant effect of app-based behavioural self-monitoring interventions in supporting improvements in both BP and medication adherence. These findings are important because they provide us with confidence that digital health interventions could be effective solutions to support health behaviour change and thus reduce BP in patients treated for hypertension during BP checks or similar clinical consultations.

This review followed the PRISMA and Cochrane guidelines with enormous population demographics from 10 countries representing LMICs. Second, a sequence of sensitivity analyses was performed to determine the strength of the pooled estimates. Third, we reported on the reduction in BP, the percentage of respondents achieving controlled BP and the odds in the intervention compared to the control. However, this review also has limitations. First, only studies published in LMICs were considered, and only two articles from Africa were included because of our inclusion/exclusion criteria: digital health interventions in only patients with hypertension but not those with comorbidities such as diabetes or HMTOD were excluded. Second, some studies were found eligible for inclusion in LMICs, but the full texts were inaccessible after several efforts to contact the corresponding authors. Third, our review was based on published data, and therefore, we did not have access to the individual patient's data; hence, we were unable to conduct multiple imputations to take into account the missing data in the individual studies. It is possible that differences in the study methods might have influenced the studies' results. Fourth, the sequence of heterogeneity examined to determine possible causes, such as subgrouping the duration of digital health intervention, year of publication and countries' economic status, could not completely explain the details of heterogeneity. Furthermore, we did not have data on access to BP-lowering medicines, socioeconomic status, unavailability of public healthcare, and knowledge of hypertension, all of which could potentially influence the results; hence, they should be taken into consideration when interpreting these findings. Other limitations were that the review did not include grey literature or unpublished studies and considered only English publications. Regarding the comparable efficiency of phone calls, SMS and smartphone app interventions, the physicians must consider digital health intervention decisions according to lifestyle modification because medication adherence was self-reported, which might have led to social desirability bias.

In conclusion, our review showed that digital health interventions may be effective in BP control, changes in lifestyle behaviours and improvements in medication adherence in LMICs. Only two studies were included in the review from Africa. The combination of digital health interventions with clinical management is crucial to achieving optimal clinical effectiveness in BP control, changes in lifestyle behaviours and improvements in medication adherence. Researchers globally should also aim to provide in detail the effectiveness and application of these interventions between patients and healthcare providers and the rate of reminders provided via digital health devices.

## Contributors

VB, AD and FA conceptualized the study. VB, AD, FA, LST and CA designed the methodology. VB, AD, FA, LST and CA performed the data collection. VB, AD, FA, LST and CA performed the data analysis. VB, AD and FA wrote the original draft of the manuscript. All authors reviewed and edited the manuscript. AD and CA supervised the data collection and analyses. VB, AD and FA performed the project administration. All authors had access to all the raw datasets. AD and CA verified the data. All authors have read and agreed to the final version of the manuscript and to its submission for publication.

## Data sharing statement

This meta-analysis used extracted data from published studies; researchers interested in obtaining data not provided in the manuscript and supplementary material can contact the corresponding authors. All extracted data are available on request from the corresponding authors.

## Declaration of interests

All authors declare no competing interests.
