# Supplementary material for: Effectiveness of digital health interventions on blood pressure control, lifestyle behaviours and adherence to medication in patients with hypertension in low-income and middle-income countries: a systematic review and meta-analysis of randomised controlled trials
Source: eClinicalMedicine. 2024 Feb 1;69:102432. doi: 10.1016/j.eclinm.2024.102432 (PMC10850120; doi:10.1016/j.eclinm.2024.102432)
Supplement: Supplementary File Figures Tables [file mmc1.docx]

**Effectiveness of digital health interventions on blood pressure control, lifestyle behaviours and adherence to medication in patients with hypertension in low-income and middle-income countries: a systematic review and meta-analysis of randomised controlled trials**

Vincent Boima^1,2†^ MD, Alfred Doku^1,2†*^ MD, Francis Agyekum^1†^ MD, Lawrence Sena Tuglo^3,4^ MPH, Charles Agyemang^2,5^ PhD

**Supplementary material**

1. Supplementary file Table 1. Search strategies of the databases (pages 1-5)
2. Supplementary file Table 2. Low-income and middle-income countries_2023_World-Bank-Country_Classification_until July 2023 (page 6)
3. Supplementary file (pages 7-16). List of studies excluded at the full-text screening stage, with brief reasons
4. Supplementary file Table 3. Characteristics of the included studies (pages 17-18)
5. Supplementary file Figure 1. Risk of bias graph of the included studies (page 18)
6. Supplementary file Figure 2. Risk of bias summary of the included studies (page 19)
7. Supplementary file Figure 3. Forest plot of the mean difference in DBP between the intervention and control groups (page 20)
8. Supplementary file Figure 4. Meta-analysis of dichotomous outcome measurements for low-salt diets (page 21)
9. Supplementary file Figure 5. Meta-analysis of dichotomous outcome measurements for PA (page 21)
10. Supplementary file Figure 6. Meta-analysis of continuous outcome measurements for weight loss (page 21)
11. Supplementary file Figure 7. Meta-analysis of dichotomous outcome measurements for BMI (page 22)
12. Supplementary file Figure 8. Meta-analysis of dichotomous outcome measurements for alcohol reduction (page 22)
13. Supplementary file Table 4. Subgroup analyses of mean differences in SBP and DBP (pages 22-23)
14. Supplementary file Figure 4. Sensitivity analyses for the SBP outcome (page 24)
15. Supplementary file Figure 5. Sensitivity analyses for the DBP outcome (page 25)
16. Supplementary file Figure 6. Funnel plot for risk of publication bias for the SBP outcome (page 26)
17. Supplementary file Figure 7. Funnel plot for risk of publication bias for the DBP outcome (page 27)

Table 1 Search strategy of the databases

Pubmed

| Concept | Search string | Records |
| --- | --- | --- |
| #1 Population | ("Hypertension"[Mesh] OR "Blood Pressure"[Mesh] OR Hypertension OR hypertensive OR "blood pressure" OR "High blood pressure" OR "elevated blood pressure" OR "abnormal blood pressure") | 938488 |
| #2 Intervention | ("Telemedicine"[Mesh] OR "Telenursing"[Mesh] OR "Telecommunications"[Mesh] OR "Remote Consultation"[Mesh] OR "Mobile Applications"[Mesh] OR "Text Messaging"[Mesh] OR "electronic health"[tiab] OR eHealth[tiab] OR e-Health[tiab] OR "mobile health"[tiab] OR mHealth[tiab] OR m-Health[tiab] OR telemedicine[tiab] OR telenursing[tiab] OR telehealth[tiab] OR teleconsultation*[tiab] OR telemonitor*[tiab] OR telecommunication*[tiab] OR tele*[tiab] "Virtual Medicine"[tiab] OR "digital health"[tiab] OR "mobile app*"[tiab] OR "mobile technolog*"[tiab] OR "mobile phone"[tiab] OR telephone[tiab] OR smartphone[tiab] OR "wireless device*"[tiab] OR "Personal Digital Assistant*"[tiab] OR "short message service"[tiab] OR "text messag*"[tiab] OR "SMS messag*"[tiab] OR "phone call*"[tiab] OR Reminder*[tiab] OR "digital intervention*"[tiab]) | 136857 |
| #3 Outcome | ("Exercise"[Mesh] OR "Weight Loss"[Mesh] OR "Diet"[Mesh] OR "Diet, Healthy"[Mesh] OR "Diet Therapy"[Mesh] OR "Dietary Approaches To Stop Hypertension"[Mesh] OR "Smoking Cessation"[Mesh] OR "Alcohol Abstinence"[Mesh] OR "Patient Compliance"[Mesh] OR "Treatment Adherence and Compliance"[Mesh] OR "Medication Adherence"[Mesh] OR "lifestyle modif*"[tiab] OR "behavior modif*"[tiab] OR "excercis*"[tiab] OR "physical activit*"[tiab] OR "weight loss"[tiab] OR "weight reduction"[tiab] OR smok*[tiab] OR alcohol*[tiab] OR "diet*"[tiab] OR "health diet"[tiab] OR "DASH Diet"[tiab] OR "medicine*"[tiab] OR medication*[tiab] OR drug*[tiab] OR "blood pressure control"[tiab] OR "blood pressure management"[tiab] OR "hypertension control"[tiab] OR "hypertension management"[tiab] OR adhere*[tiab] OR complian*[tiab] OR noncomplan*[tiab] OR nonadhere*[tiab] OR concord*[tiab] OR "persist*"[tiab]) | 5491979 |
| #4 Study design | ("Randomized Controlled Trial" [Publication Type] OR "Randomized Controlled Trials as Topic"[Mesh] OR Controlled Trial*[tiab] OR RCT[tiab] OR randomiz*[tiab] OR randomis*[tiab] OR Random*[tiab]) | 1649697 |
|  | #1 AND #2 AND #3 AND #4 | 1281 |
|  | Publication date: 2009 - 2023 | 1,044 |

Scopus

| Concept | Search string | Results |
| --- | --- | --- |
| #1 Population | TITLE-ABS-KEY ( hypertension OR "blood pressure" OR "high blood pressure" OR "elevated blood pressure" OR "abnormal blood pressure" OR hypertensive ) | 1403733 |
| #2 Intervention | TITLE-ABS-KEY ( telemedicine OR telenursing OR telecommunication* OR telehealth OR "teleconsultation*" OR "tele-education" OR telemonitor* OR "Remote Consultation*" OR "Virtual Medicine" OR "digital health" OR "electronic health" OR ehealth OR e-health OR "mobile health" OR mhealth OR m-health OR "telephone-based" OR "phone-based" OR "mobile technolog*" OR "Mobile App*" OR "mobile phone" OR "telephone" OR "smartphone" OR "wireless device*" OR "Personal Digital Assistant*" OR "short message service" OR "SMS" OR "text messag*" OR "phone call*" OR reminder* OR "digital intervention*" ) | 1094775 |
| #3 Outcome | TITLE-ABS-KEY ( exercise OR "physical activit*" OR "weight loss" OR "weight reduction" OR "weight therap*" OR smok* OR "smoking cessation" OR alcohol* OR "alcohol retraction" OR diet* OR "healthy diet" OR "dietary therap*" OR "Dietary Approaches To Stop Hypertension" OR "DASH diet" OR "medication adherence" OR medicine* OR medication* OR drug* OR "blood pressure control" OR "blood pressure management" OR "hypertension control" OR "hypertension management" OR adhere* OR complian* OR noncomplian* OR nonadhere* OR concord* OR persist* ) | 15475964 |
| #4 Study design | TITLE-ABS-KEY ( "randomized controlled trial*" OR "controlled trial*" OR "RCT*" OR randomiz* OR randomis* OR random* ) | 3160828 |
|  | #1 AND #2 AND #3 AND #4 | 2793 |
|  | Publication date: 2009 - 2023 | 2417 |

Web of science

| Concept | Search string | Results |
| --- | --- | --- |
| #1 Population | TS=( hypertension OR "blood pressure" OR "high blood pressure" OR "elevated blood pressure" OR "abnormal blood pressure" OR hypertensive ) | 822088 |
| #2 Intervention | TS=( telemedicine OR telenursing OR telecommunication* OR telehealth OR "teleconsultation*" OR "tele-education" OR telemonitor* OR "Remote Consultation*" OR "Virtual Medicine" OR "digital health" OR "electronic health" OR ehealth OR e-health OR "mobile health" OR mhealth OR m-health OR "telephone-based" OR "phone-based" OR "mobile technolog*" OR "Mobile App*" OR "mobile phone" OR "telephone" OR "smartphone" OR "wireless device*" OR "Personal Digital Assistant*" OR "short message service" OR "SMS" OR "text messag*" OR "phone call*" OR reminder* OR "digital intervention*" ) | 407352 |
| #3 Outcome | TS=( exercise OR "physical activit*" OR "weight loss" OR "weight reduction" OR "weight therap*" OR smok* OR "smoking cessation" OR alcohol* OR "alcohol retraction" OR diet* OR "healthy diet" OR "dietary therap*" OR "Dietary Approaches To Stop Hypertension" OR "DASH diet" OR "medication adherence" OR medicine* OR medication* OR drug* OR "blood pressure control" OR "blood pressure management" OR "hypertension control" OR "hypertension management" OR adhere* OR complian* OR noncomplian* OR nonadhere* OR concord* OR persist* ) | 6816187 |
| #4 Study design | TS=( "randomized controlled trial*" OR "controlled trial*" OR RCT* OR randomiz* OR randomis* OR random* ) | 2431498 |
|  | #1 AND #2 AND #3 AND #4 | 1922 |
|  | Publication date: 2009 - 2023 | 1646 |

Embase

| Concept | Search string | Results |
| --- | --- | --- |
| #1 Population | (exp hypertension/ or exp elevated blood pressure/ or exp abnormal blood pressure/ or exp blood pressure/ or hypertens*.ti,ab,kf. or "high blood pressure".ti,ab,kf. or "elevated blood pressure".ti,ab,kf. or "abnormal blood pressure".ti,ab,kf. or "blood pressure".ti,ab,kf.) | 1747523 |
| #2 Intervention | (exp telemedicine/ or exp telenursing/ or exp telehealth/ or exp teleconsultation/ or exp telecommunication/ or exp mobile health application/ or exp mobile application/ or exp mobile phone/ or exp telephone/ or exp personal digital assistant/ or telemedicine.ti,ab,kf. or telenursing.ti,ab,kf. or telehealth.ti,ab,kf. or teleconsultation.ti,ab,kf. or telecommunication.ti,ab,kf. or tele*.ti,ab,kf. or "remote consultation".ti,ab,kf. or "electronic health".ti,ab,kf. or ehealth.ti,ab,kf. or e-health: .ti,ab,kf. or "mobile health".ti,ab,kf. or mhealth.ti,ab,kf. or m-Health.ti,ab,kf. or teleeducation.ti,ab,kf. or telemonitor*.ti,ab,kf. or "virtual Medicine".ti,ab,kf. or "digital health".ti,ab,kf. or mobile app*.ti,ab,kf. or "mobile technolog*".ti,ab,kf. or "mobile phone".ti,ab,kf. or telephone.ti,ab,kf. or smartphone.ti,ab,kf. or "wireless device*".ti,ab,kf. or "Personal Digital Assistant*".ti,ab,kf. or "short message service".ti,ab,kf. or "text messag*".ti,ab,kf. or "SMS".ti,ab,kf. or Reminder*.ti,ab,kf. or "digital intervention*".ti,ab,kf.) | 465105 |
| #3 Outcome | (exp exercise/ or exp physical activity/ or exp weight loss program/ or exp diet/ or exp healthy diet/ or exp DASH diet/ or exp smoking/ or exp smoking cessation/ or exp smoking reduction/ or exp alcohol abstinence/ or exp alcohol/ or exp blood pressure regulation/ or exp medication compliance/ or "blood pressure control".ab,ti,kf or "blood pressure management".ti,ab,kf. or "hypertension control".ti,ab,kf. or "hypertension management".ti,ab,kf. or exercis*.ti,ab,kf. or "physical activit*".ti,ab,kf. or "weight reduction".ti,ab,kf. or " weight therap*".ti,ab,kf. or diet*.ti,ab,kf. or "healthy diet".ti,ab,kf. or "diet* therap*".ti,ab,kf. or "Dietary Approaches To Stop Hypertension".ti,ab,kf. or "DASH diet".ti,ab,kf. OR smok*.ti,ab,kf. or alcohol*.ti,ab,kf. or medication*.ti,ab,kf. or adhere*.ti,ab,kf. or complian*.ti,ab,kf. or noncomplan*.ti,ab,kf. or nonadhere*.ti,ab,kf. or concord*.ti,ab,kf. or persist*.ti,ab,kf.) | 4837063 |
| #4 Study design | (exp randomized controlled trial/ or "controlled trial*".ti,ab,kf. or RCT*.ti,ab,kf. or randomiz*.ti,ab,kf. or randomis.ti,ab,kf. or random*.ti,ab,kf.) | 2118787 |
|  | #1 AND #2 AND #3 AND #4 | 3121 |
|  | Publication date: 2009 - 2023 | 2818 |

CINAHL

| Concept | Search string | Results |
| --- | --- | --- |
| #1 Population | (MH "Hypertension+" OR MH "Blood Pressure+") OR TI("hypertens*" OR "elevated blood pressure" OR "abnormal blood pressure" OR "high blood pressure" OR "blood pressure") OR AB("hypertens*" OR "elevated blood pressure" OR "abnormal blood pressure" OR "high blood pressure" OR "blood pressure") OR ("hypertens*" OR "elevated blood pressure" OR "abnormal blood pressure" OR "high blood pressure" OR "blood pressure") | 217,337 |
| #2 Intervention | (MH "Telemedicine+" OR MH "Telenursing+" OR MH "Telehealth+" OR MH "Telecommunications+" OR MH "Remote Consultation+" OR MH "Telephone+" OR MH "Mobile Applications" OR MH "Social Media+ ) OR TI(Telemedicine OR Telenursing OR Telehealth OR Telecommunication OR Teleeducation OR Telemonitr* OR Tele* "Remote consultation*" OR "electronic health" OR eHealth OR e-Health OR "mobile health" OR mHealth OR m-Health OR "digital health" OR "digital intervention*" OR "virtual medicine" OR "mobile app*" OR "mobile technolog*" OR "mobile phone" OR "telephone" OR "Smart phone" OR "wireless device" OR "Personal Digital Assistant" OR "short message service" OR SMS OR "text messag*" OR reminder*) OR AB(Telemedicine OR Telenursing OR Telehealth OR Telecommunication OR Teleeducation OR Telemonitr* OR Tele* "Remote consultation*" OR "electronic health" OR eHealth OR e-Health OR "mobile health" OR mHealth OR m-Health OR "digital health" OR "digital intervention*" OR "virtual medicine" OR "mobile app*" OR "mobile technolog*" OR "mobile phone" OR "telephone" OR "Smart phone" OR "wireless device" OR "Personal Digital Assistant" OR "short message service" OR SMS OR "text messag*" OR reminder*) OR (Telemedicine OR Telenursing OR Telehealth OR Telecommunication OR Teleeducation OR Telemonitr* OR Tele* "Remote consultation*" OR "electronic health" OR eHealth OR e-Health OR "mobile health" OR mHealth OR m-Health OR "digital health" OR "digital intervention*" OR "virtual medicine" OR "mobile app*" OR "mobile technolog*" OR "mobile phone" OR "telephone" OR "Smart phone" OR "wireless device" OR "Personal Digital Assistant" OR "short message service" OR SMS OR "text messag*" OR reminder*) | 178,094 |
| #3 Outcome | (MH "Exercise+" OR MH "Physical Activity" OR MH "Weight Reduction Programs" OR MH "Diet+" OR MH "Diet Therapy+" OR MH "DASH Diet" OR MH "Smoking+ OR MH "Smoking Cessation" OR MH "Alcohol Drinking+" OR MH "Medication Compliance") OR TI("exercise*" OR "physical activit*" OR "weight reduction" OR "weight therap*" OR "diet*" OR "healthy diet" OR "diet* therap*" OR "Dietary Approaches To Stop Hypertension" OR "DAHS diet" OR "smok*" OR "Alcohol*" OR "medication*" OR "medication adherence" OR "adhere*" OR "nonadhere*" OR "complian*" OR "noncomplain*" OR "concord*" OR "persist*") OR AB("exercise*" OR "physical activit*" OR "weight reduction" OR "weight therap*" OR "diet*" OR "healthy diet" OR "diet* therap*" OR "Dietary Approaches To Stop Hypertension" OR "DAHS diet" OR "smok*" OR "Alcohol*" OR "medication*" OR "medication adherence" OR "adhere*" OR "nonadhere*" OR "complian*" OR "noncomplain*" OR "concord*" OR "persist*") OR ("exercise*" OR "physical activit*" OR "weight reduction" OR "weight therap*" OR "diet*" OR "healthy diet" OR "diet* therap*" OR "Dietary Approaches To Stop Hypertension" OR "DAHS diet" OR "smok*" OR "Alcohol*" OR "medication*" OR "medication adherence" OR "adhere*" OR "nonadhere*" OR "complian*" OR "noncomplain*" OR "concord*" OR "persist*") | 295,521 |
| #4 Study deign | (MH "Randomized Controlled Trials+" OR MH "Clinical Trials+) OR TI("randomized controlled trial*" OR "controlled trial*" OR "RCT*" OR "Random*") OR AB("randomized controlled trial*" OR "controlled trial*" OR "RCT*" OR "Random*") OR ("randomized controlled trial*" OR "controlled trial*" OR "RCT*" OR "Random*") | 137,901 |
|  | #1 AND #2 AND #3 AND #4 | 80 |
|  | Publication date: 2009 - 2023 | 79 |

Cochrane Central Register of Controlled Trials Search

| Concept | Search string | Results |
| --- | --- | --- |
| #1 Population | Hypertension OR Blood Pressure OR hypertensive OR High blood pressure OR elevated blood pressure OR abnormal blood pressure | 168212 |
| #2 Intervention | Telemedicine OR Telenursing OR Telecommunications OR Remote Consultation OR Mobile Applications OR Text Messaging OR electronic health OR eHealth OR e-Health OR mobile health OR mHealth OR m-Health OR telemedicine OR telenursing OR telehealth OR teleconsultation* OR telemonitor* OR telecommunication* OR tele* Virtual Medicine OR digital health OR mobile app* OR mobile technolog* OR mobile phone OR telephone OR smartphone OR wireless device* OR Personal Digital Assistant* OR short message service OR text messag* OR SMS messag* OR phone call* OR Reminder* OR digital intervention* | 95146 |
| #3 Outcome | Exercise OR Weight Loss OR Diet OR Diet, Healthy OR Diet Therapy OR Dietary Approaches To Stop Hypertension OR Smoking Cessation OR Alcohol Abstinence OR Patient Compliance OR Treatment Adherence and Compliance OR Medication Adherence OR lifestyle modif* OR behavior modif* OR excercis* OR physical activit* OR weight loss OR weight reduction OR smok* OR alcohol* OR diet* OR health diet OR DASH Diet OR medicine* OR medication* OR drug* OR blood pressure control OR blood pressure management OR hypertension control OR hypertension management OR adhere* OR complian* OR noncomplan* OR nonadhere* OR concord* OR persist* | 1191142 |
| #4 Study deign | Randomized Controlled Trials OR Clinical Trials OR randomized controlled trial* OR controlled trial* OR RCT* OR Random* OR RCT OR randomiz* OR randomis* | 2047858 |
|  | #1 AND #2 AND #3 AND #4 | 11476 |
|  | Publication date: 2009 - 2023 | 6827 |
|  | in Trials with Hypertension in Cochrane Groups | 1311 |

Table 2 Low-income and middle-income countries_2023_World-Bank-Country_Classification_until-July-2023

| Keyword | Synonyms |
| --- | --- |
| Low-income and middle-income countries | "low income country" OR “lower income country OR “LIC” OR "low income countries" OR “lower income countries” OR “LICs” OR "middle income country" OR “MIC” OR "middle income countries" OR “MICs” OR “lower middle income country" OR “LIMC” OR “lower middle income countries" OR "LMICs" OR "developing country" OR "developing countries” OR “developing nation” OR “developing nations” OR "less developed country" OR "less developed countries" OR “Afghanistan” OR “Albania” OR “Algeria” OR “American Samoa” OR “Angola” OR "Antigua and Barbuda" OR “Argentina” OR “Armenia” OR “Azerbaijan” OR “Bahrain” OR “Bangladesh” OR “Barbados” OR “Benin” OR “Byelarus” OR “Belize” OR “Bhutan” OR “Bolivia” OR “Bosnia-Herzegovina” OR “Botswana” OR “Brazil” OR “Bulgaria” OR “Burkina Faso” OR “Burundi” OR “Cambodia” OR “Cameroon” OR “Cape Verde” OR “Central African Republic” OR “Chad” OR “China” OR “Colombia” OR “Comoros” OR “Congo” OR “Costa Rica” OR “Cote d'Ivoire” OR “Croatia” OR “Cuba” OR “Cyprus” OR “Czechoslovakia” OR “Czech Republic” OR “Slovakia” OR “Djibouti” OR "Democratic Republic of the Congo" OR “Dominica” OR “Dominican Republic” OR “East Timor” OR “Ecuador” OR “Egypt” OR “El Salvador” OR “Eritrea” OR “Estonia” OR “Ethiopia” OR “Fiji” OR “Gabon” OR “Gambia” OR "Georgia (Republic)" OR “Ghana” OR “Greece” OR “Grenada” OR “Guatemala” OR “Guinea” OR “Guinea-Bissau” OR “Guam” OR “Guyana” OR “Haiti” OR “Honduras” OR “Hungary” OR “India” OR “Indonesia” OR “Iran” OR “Iraq” OR “Jamaica” OR “Jordan” OR “Kazakhstan” OR “Kenya” OR “Korea” OR “Kosovo” OR “Kyrgyzstan” OR “Laos” OR “Latvia” OR “Lebanon” OR “Lesotho” OR “Liberia” OR “Libya” OR “Lithuania” OR “Macedonia” OR “Madagascar” OR “Malaysia” OR “Malawi” OR “Mali” OR “Malta” OR “Mauritania” OR “Mauritius” OR “Mexico” OR “Micronesia” OR “Middle East” OR “Moldova” OR “Mongolia” OR “Montenegro” OR “Morocco” OR “Mozambique” OR “Montenegro” OR “Myanmar” OR “Namibia” OR “Nepal” OR “Netherlands Antilles” OR “New Caledonia” OR “Nicaragua” OR “Niger” OR “Nigeria” OR “Oman” OR “Pakistan” OR “Palau” OR “Panama” OR “Papua New Guinea” OR “Paraguay” OR “Peru” OR “Philippines” OR “Poland” OR “Portugal” OR “Puerto Rico” OR “Romania” OR “Russia” OR “Rwanda” OR "Saint Kitts and Nevis" OR “Saint Lucia” OR "Saint Vincent and the Grenadines" OR “Samoa” OR “Saudi Arabia” OR “Senegal” OR “Serbia” OR “Seychelles” OR “Sierra Leone” OR “Slovenia” OR “Sri Lanka” OR “Somalia” OR “South Africa” OR “Sudan” OR “Suriname” OR “Swaziland” OR “Syria” OR “Tajikistan” OR “Tanzania” OR “Thailand” OR “Togo” OR “Tonga” OR "Trinidad and Tobago" OR “Tunisia” OR “Turkey” OR “Turkmenistan” OR “Uganda” OR “Ukraine” OR “Uruguay” OR “USSR” OR “Uzbekistan” OR “Vanuatu” OR “Venezuela” OR “Vietnam” OR “Yemen” OR “Yugoslavia” OR “Zambia” OR “Zimbabwe” |
|  | "randomised controlled trial" OR "controlled clinical trial" OR “randomized clinical trial” OR “randomized comparative trial” OR “randomized control trial” OR “randomized controlled clinical trial” OR "randomised trial" OR “trial” OR “randomised” OR “clinical trial” |

**List of studies excluded at the full-text screening stage, with brief reasons**

**High-income countries**

1. Davidson TM, McGillicuddy J, Mueller M, et al. Evaluation of an mHealth medication regimen self-management program for African American and Hispanic uncontrolled hypertensives. J Pers Med 2015; 5: 389–405
2. Mehta SJ, Volpp KG, Troxel AB, et al. Electronic pill bottles or bidirectional text messaging to improve hypertension medication adherence (Way 2 Text): a randomized clinical trial. J Gen Intern Med 2019; 34: 2397–404.
3. Schroeder EB, Moore KR, Manson SM, et al. A randomized clinical trial of an interactive voice response and text message intervention for individuals with hypertension. J Clin Hypertens (Greenwich) 2020; 22: 1228–38.
4. Zahr RS, Anthony CA, Polgreen PM, et al. A texting-based blood pressure surveillance intervention. J Clin Hypertens (Greenwich) 2019; 21: 1463–70.
5. Chandler J, Sox L, Diaz V, et al. Impact of 12-month smartphone breathing meditation program upon systolic blood pressure among non-medicated stage 1 hypertensive adults. Int J Environ Res Public Health 2020; 17: 1955
6. Chandler J, Sox L, Kellam K, Feder L, Nemeth L, Treiber F. Impact of a culturally tailored mHealth medication regimen self-management program upon blood pressure among hypertensive Hispanic adults. Int J Environ Res Public Health 2019; 16: 1226
7. Cho SMJ, Lee JH, Shim JS, et al. Effect of smartphone-based lifestyle coaching app on community-dwelling population with moderate metabolic abnormalities: randomized controlled trial. J Med Internet Res 2020; 22: e17435.
8. Dorsch MP, Cornellier ML, Poggi AD, et al. Effects of a novel contextual just-in-time mobile app intervention (LowSalt4Life) on sodium intake in adults with hypertension: pilot randomized controlled trial. JMIR Mhealth Uhealth 2020; 8: e1669
9. Frias J, Virdi N, Raja P, Kim Y, Savage G, Osterberg L. Effectiveness of digital medicines to improve clinical outcomes in patients with uncontrolled hypertension and type 2 diabetes: prospective, open-label, cluster-randomized pilot clinical trial. J Med Internet Res 2017; 19: e246
10. Moore JO, Marshall MA, Judge DC, et al. Technology-supported apprenticeship in the management of hypertension: a randomized controlled trial. J Clin Outcomes Manag 2014; 21: 110–12.
11. Morawski K, Ghazinouri R, Krumme A, et al. Association of a smartphone application with medication adherence and blood pressure control: the MedISAFE-BP randomized clinical trial. JAMA Intern Med 2018; 178: 802–09
12. Or CK, Liu K, So MKP, et al. Improving self-care in patients with coexisting type 2 diabetes and hypertension by technological surrogate nursing: randomized controlled trial. J Med Internet Res 2020; 22: e16769
13. Petrella RJ, Stuckey MI, Shapiro S, Gill DP. Mobile health, exercise and metabolic risk: a randomized controlled trial. BMC Public Health 2014; 14: 1082
14. Yun YH, Kang E, Cho YM, et al. Efficacy of an electronic health management program for patients with cardiovascular risk: randomized controlled trial. J Med Internet Res 2020; 22: e15057
15. Bove AA, Homko CJ, Santamore WP, Kashem M, Kerper M, Elliott DJ. Managing hypertension in urban underserved subjects using telemedicine—a clinical trial. Am Heart J 2013; 165: 615–21
16. Bray EP, Jones MI, Banting M, et al. Performance and persistence of a blood pressure self-management intervention: telemonitoring and self-management in hypertension (TASMINH2) trial. J Hum Hypertens 2015; 29: 436–41
17. Kao CW, Chen TY, Cheng SM, Lin WS, Chang YC. A web-based self-titration program to control blood pressure in patients with primary hypertension: randomized controlled trial. J Med Internet Res 2019; 21: e15836
18. Nolan RP, Feldman R, Dawes M, et al. Randomized controlled trial of e-counseling for hypertension: REACH. Circ Cardiovasc Qual Outcomes 2018; 11: e004420
19. Thiboutot J, Sciamanna CN, Falkner B, et al. Effects of a web-based patient activation intervention to overcome clinical inertia on blood pressure control: cluster randomized controlled trial. J Med Internet Res 2013; 15: e158

**Ineligible outcomes**

1. Baer, H. J., et al. (2020). "Effect of an Online Weight Management Program Integrated With Population Health Management on Weight Change: A Randomized Clinical Trial." JAMA: Journal of the American Medical Association 324(17): 1737-1746.
2. Basudev N, et al. (2016). "A prospective randomized controlled study of a virtual clinic integrating primary and specialist care for patients with Type 2 diabetes mellitus." 33(6): 768.
3. Brath H, et al. (2013). "Mobile health (mHealth) based medication adherence measurement - a pilot trial using electronic blisters in diabetes patients." 76 Suppl 1: 47.
4. Cesa, G. L., et al. (2011). "TECNOB study: Ad interim results of a randomized controlled trial of a multidisciplinary telecare intervention for obese patients with type-2 diabetes." Clinical Practice and Epidemiology in Mental Health 7: 44-50.
5. Christison-Lagay, J., et al. (2010). "Managing the space between visits: A randomized trial of disease management for diabetes in a community health center." Journal of General Internal Medicine 25(10): 1116-1122.
6. Cho J, et al. (2011). "Effectiveness and safety of a glucose data-filtering system with automatic response software to reduce the physician workload in managing type 2 diabetes." 17(5): 257.
7. Del Prato S, et al. (2012). "Telecare Provides comparable efficacy to conventional self-monitored blood glucose in patients with type 2 diabetes titrating one injection of insulin glulisine-the ELEONOR study." 14(2): 175.
8. Diantini, A., et al. (2020). "Collaboration between interprofessional healthcare and patients to improve quality of life type 2 diabetes via smartphone application." International Journal of Pharmaceutical Research 13(1): 645-650.
9. Fischer H et al. (2012). "Nurse-run, telephone-based outreach to improve lipids in people with diabetes." 18(2): 77.
10. Glasgow RE, et al. (2012) “Twelve-month outcomes of an internet-based diabetes self-management support program.” Patient Education Counselling 87: 81-92.
11. Goode A et al. (2015). "Relationship between intervention dose and outcomes in living well with diabetes--a randomized trial of a telephone-delivered lifestyle-based weight loss intervention." 30(2): 120.
12. Hansen, C. R., et al. (2017). "Video consultations as add-on to standard care among patients with type 2 diabetes not responding to standard regimens: a randomized controlled trial." European journal of endocrinology 176(6): 727-736.
13. Holland-Carter, L., et al. (2017). "Impact on psychosocial outcomes of a nationally available weight management program tailored for individuals with type 2 diabetes: Results of a randomized controlled trial." Journal of Diabetes & its Complications 31(5): 891-897.
14. Ibrahim M, T. H. (2019). "The ABCs outcomes after using a smart phone-based lifestyle application in subjects with type 2 diabetes." 13(2): 55.
15. Kanadli, K. A., et al. (2016). "Does Telephone Follow-Up and Education Affect Self-Care and Metabolic Control in Diabetic Patients?" Holistic Nursing Practice 30(2): 70-77.
16. Kardas, P., et al. (2016). "Type 2 Diabetes Patients Benefit from the COMODITY12 mHealth System: Results of a Randomised Trial." Journal of Medical Systems 40(12): 259.
17. Khunti, K., et al. (2021). "Promoting physical activity in a multi-ethnic population at high risk of diabetes: the 48-month PROPELS randomised controlled trial." BMC Medicine 19(1): 130.
18. Kumar, D., et al. (2018). "Effectiveness of randomized control trial of mobile phone messages on control of fasting blood glucose in patients with type-2 diabetes mellitus in a Northern State of India." Indian Journal of Public Health 62(3): 224-226.
19. Levy, N., et al. (2015). "The Mobile Insulin Titration Intervention (MITI) for Insulin Adjustment in an Urban, Low-Income Population: Randomized Controlled Trial." Journal of Medical Internet Research 17(7): e180.
20. Li, C. (2018). "The effect of case management model based on mobile health on selfmanagement and blood glucose control of type 2 diabetes patients." 9: 141.
21. Logan, A. G., et al. (2012). "Effect of home blood pressure telemonitoring with self-care support on uncontrolled systolic hypertension in diabetics." Hypertension (0194911X) 60(1): 51-57.
22. Luley, C., et al. (2011). "Weight loss in obese patients with type 2 diabetes: Effects of telemonitoring plus a diet combination - The Active Body Control (ABC) Program." Diabetes Research & Clinical Practice 91(3): 286-292.
23. Muralidharan S, et al. (2019). "Engagement and Weight Loss: results from the Mobile Health and Diabetes Trial." 21(9): 507.
24. O'Neil, P. M., et al. (2016). "Randomized controlled trial of a nationally available weight control program tailored for adults with type 2 diabetes." Obesity (19307381) 24(11): 2269-2277.
25. Owolabi E et al. (2019). "Efficacy, acceptability and feasibility of daily text-messaging in promoting glycaemic control and other clinical outcomes in a low-resource setting of South Africa: a randomised controlled trial." 14(11): e0224791.
26. Patnaik L, et al. (2014). "Mobile based intervention for reduction of coronary heart disease risk factors among patients with diabetes mellitus attending a tertiary care hospital of India." 5(4): 28.
27. Plotnikoff, R. C., et al. (2017). "Integrating smartphone technology, social support and the outdoor physical environment to improve fitness among adults at risk of, or diagnosed with, Type 2 Diabetes: Findings from the 'eCoFit' randomized controlled trial." Preventive Medicine 105: 404-411.
28. Poppe L, et al. (2019). "Efficacy of a Self-Regulation-Based Electronic and Mobile Health Intervention Targeting an Active Lifestyle in Adults Having Type 2 Diabetes and in Adults Aged 50 Years or Older: two Randomized Controlled Trials." 21(8): e13363.
29. Rasmussen O et al. (2016). "Telemedicine compared with standard care in type 2 diabetes mellitus: a randomized trial in an outpatient clinic." 22(6): 363.
30. Redfern, J., et al. (2020). "A digital health intervention for cardiovascular disease management in primary care (CONNECT) randomized controlled trial." npj Digital Medicine 3(1): 117.
31. Ritchie N et al. (2020). "Supplemental Text Message Support with the National Diabetes Prevention Program: pragmatic Comparative Effectiveness Trial." 8(6): e15478.
32. Sani M, et al. (2018). "Effect of telemedicine messages integrated with peer group support on glycemic control in type 2 diabetics, Kingdom of Saudi Arabia." 38(4): 495.
33. Schillinger, D., et al. (2009). "Effects of self-management support on structure, process, and outcomes among vulnerable patients with diabetes." Diabetes Care 32(4): 559-566.
34. Shea, S., et al. (2009). "A randomized trial comparing telemedicine case management with usual care in older, ethnically diverse, medically underserved patients with diabetes mellitus: 5 year results of the IDEATel study." Journal of the American Medical Informatics Association 16(4): 446-456.
35. Taniguchi S, et al. (2017). "Efficacy of PHR integrated with EHR and self-monitoring devices on self-care in patients with type 2 diabetes." 66: A613.
36. Tekkesin, A. I., et al. (2021). "Lifestyle intervention using mobile technology and smart devices in patients with high cardiovascular risk: A pragmatic randomised clinical trial." Atherosclerosis 319: 21-27.
37. Vaughan, E. M., et al. (2021). "A Telehealth-supported, Integrated care with CHWs, and MEdication-access (TIME) Program for Diabetes Improves HbA1c: a Randomized Clinical Trial." Journal of General Internal Medicine 36(2): 455-463.
38. Wakefield B et al. (2011). "Effectiveness of home telehealth in comorbid diabetes and hypertension: a randomized, controlled trial." 17(4): 254.
39. Wakefield B et al. (2014). "Effect of home telemonitoring on glycemic and blood pressure control in primary care clinic patients with diabetes." 20(3): 199.
40. Wang, X., et al. (2020). "The role of text messaging intervention in Inner Mongolia among patients with type 2 diabetes mellitus: a randomized controlled trial." BMC medical informatics and decision
41. Weinstock R, et al. (2011). "Lessened decline in physical activity and impairment of older adults with diabetes with telemedicine and pedometer use: results from the IDEATel study." 40(1): 98.
42. Wild S et al. (2016). "Supported Telemonitoring and Glycemic Control in People with Type 2 Diabetes: the Telescot Diabetes Pragmatic Multicenter Randomized Controlled Trial." 13(7): e1002098.
43. Yasmin F et al. (2020). “The influence of mobile phone-based health reminders on patient adherence to medications and healthy lifestyle recommendations for effective management of diabetes type 2: a randomized control trial in Dhaka, Bangladesh” BMC Health Serv Res 20(1): 520.
44. Young H, et al. (2020). "Nurse Coaching and Mobile Health Compared With Usual Care to Improve Diabetes Self-Efficacy for Persons With Type 2 Diabetes: randomized Controlled Trial." 8(3): e16665.

**Ineligible population**

1. Apiñaniz, A., et al. (2019). "Effectiveness of randomized controlled trial of a mobile app to promote healthy lifestyle in obese and overweight patients." Family Practice 36(6): 699-705.
2. Azar, K. M. J., et al. (2015). "Virtual small groups for weight management: an innovative delivery mechanism for evidence-based lifestyle interventions among obese men." Translational Behavioral Medicine 5(1): 37-44.
3. Befort, C. A., et al. (2021). "Effect of Behavioral Therapy with In-Clinic or Telephone Group Visits vs In-Clinic Individual Visits on Weight Loss among Patients with Obesity in Rural Clinical Practice: A Randomized Clinical Trial." JAMA - Journal of the American Medical Association 325(4): 363-372.
4. Bennett G et al. (2018). "Effectiveness of an App an d Provider Counseling for Obesity Treatment in Primary Care." 55(6): 777.
5. Block G et al. (2015). “Diabetes Prevention and Weight Loss with a Fully Automated Behavioral Intervention by Email, Web, and Mobile Phone: A Randomized Controlled Trial Among Persons with Prediabetes” J Med Internet Res 17(10): e240.
6. Crowley, M. J., et al. (2016). "Practical Telemedicine for Veterans with Persistently Poor Diabetes Control: A Randomized Pilot Trial." Telemedicine journal and e-health: the official journal of the American Telemedicine Association 22(5): 376-384.
7. Foley P, et al. (2012). "Weight gain prevention among black women in the rural community health center setting: the Shape Program." 12: 305.
8. Fukuoka, Y., et al. (2015). "A novel diabetes prevention intervention using a mobile app: A randomized controlled trial with overweight adults at risk." American Journal of Preventive Medicine 49(2): 223-237.
9. Jassal S et al. (2017). "An integrated smartphone application improves patient safety and intervention adherence: a randomized controlled trial (RCT) with an active control group." 28: B6.
10. Kelders, S. M., et al. (2011). "Effectiveness of a Web-based Intervention Aimed at Healthy Dietary and Physical Activity Behavior: A Randomized Controlled Trial About Users and Usage." Journal of Medical Internet Research 13(2): e32-e32.
11. Kempf K, et al. (2018). "Telemedical coaching improves long-term weight loss in overweight persons: a randomized controlled trial." 2018(no pagination).
12. Laing, B. Y., et al. (2014). "Effectiveness of a smartphone application for weight loss compared with usual care in overweight primary care patients: a randomized, controlled trial." Annals of Internal Medicine 161: S5-S12.
13. Lee C et al. (2018). "Mobile health, physical activity, and obesity: subanalysis of a randomized controlled trial." 97(38): e12309.
14. Lilholt, P. H., et al. (2015). "Heuristic evaluation of a telehealth system from the Danish TeleCare North Trial." International Journal of Medical Informatics 84(5): 319-326.
15. Little P, et al. (2016). "An internet-based intervention with brief nurse support to manage obesity in primary care (POWeR+): a pragmatic, parallel-group, randomised controlled trial." 4(10): 821.
16. Louis Walthouwer, M. J., et al. (2015). "Use and Effectiveness of a Video- and Text-Driven Web-Based Computer-Tailored Intervention: Randomized Controlled Trial." Journal of Medical Internet Research 17(9): e222-213.
17. McLeod M et al. (2020). “Impact of a comprehensive digital health programme on HbA1c and weight after 12 months for people with diabetes and prediabetes: a randomised controlled trial.” Diabetologia 63(12): 2559.
18. Miller, K. M., et al. (2018). "Total lifestyle coaching: A pilot study evaluating the effectiveness of a mind-body and nutrition telephone coaching program for obese adults at a community health center."
19. Nanditha A et al. (2020). “A pragmatic and scalable strategy using mobile technology to promote sustained lifestyle changes to prevent type 2 diabetes in India and the UK: a randomised controlled trial.” Diabetologia 63(3): 486.
20. Rosas, L. G., et al. (2020). "Effect of a Culturally Adapted Behavioral Intervention for Latino Adults on Weight Loss Over 2 Years: A Randomized Clinical Trial." JAMA Network Open 3(12): e2027744-e2027744.
21. Rossi, M. C., et al. (2010). "An interactive diary for diet management (DAI): a new telemedicine system able to promote body weight reduction, nutritional education, and consumption of fresh local produce." Diabetes Technology & Therapeutics 12(8): 641-647.
22. Rubinstein A et al. (2016). “Effectiveness of an mHealth intervention to improve the cardiometabolic profile of people with prehypertension in low-resource urban settings in Latin America: a randomised controlled trial” Lancet Diabetes Endocrinol 4(1): 52.
23. Steinberg, D. M., et al. (2014). "Adherence to self-monitoring via interactive voice response technology in an eHealth intervention targeting weight gain prevention among Black women: randomized controlled trial." Journal of Medical Internet Research 16(4): e114-111.
24. Svetkey, L. P., et al. (2014). "Greater weight loss with increasing age in the weight loss maintenance trial." Obesity (19307381) 22(1): 39-44.
25. Tarraga M et al. (2017). "Application of telemedicine in obesity management." L'application de la telemedecine dans la prise en charge de l'obesite 6(1): 3.
26. Vedanthan R, et al. (2019). "Community Health Workers Improve Linkage to Hypertension Care in Western Kenya." J Am Coll Cardiol 74(15): 1897.
27. Ventura M et al. (2019). "A Pilot Randomized Controlled Trial of a Telenutrition Weight Loss Intervention in Middle-Aged and Older Men with Multiple Risk Factors for Cardiovascular Disease." 11(2).
28. Von Storch K et al. (2019). “Telemedicine-assisted self-management program for type 2 diabetes patients.” Diabetes Technol Ther 21(9): 514-521.
29. Vuorinen, A.L., et al. (2014). "Use of home telemonitoring to support multidisciplinary care of heart failure patients in Finland: randomized controlled trial." Journal of Medical Internet Research 16(12): e282-e282.
30. Whitelock, V., et al. (2019). "A smartphone based attentive eating intervention for energy intake and weight loss: results from a randomised controlled trial." BMC Public Health 19(1): 1-11.
31. Zheng X, et al. (2019). "Effect of Text Messaging on Risk Factor Management in Patients With Coronary Heart Disease: the CHAT Randomized Clinical Trial." 12(4): e005616.
32. Argay M, Meskó A, Zelkó R, Hankó B. Therapy reminder message for Hungarian patients with type 2 diabetes. Acta Pol Pharm 2015; 72: 1289–93
33. Arora S, Peters AL, Burner E, Lam CN, Menchine M. Trial to examine text message-based mHealth in emergency department patients with diabetes (TExT-MED): a randomized controlled trial. Ann Emerg Med 2014; 63: 745–54.e6
34. Dobson R, Whittaker R, Jiang Y, et al. Long-term follow-up of a randomized controlled trial of a text-message diabetes self-management support programme, SMS4BG. Diabet Med 2020; 37: 311–18
35. Fang R, Deng X. Electronic messaging intervention for management of cardiovascular risk factors in type 2 diabetes mellitus: a randomised controlled trial. J Clin Nurs 2018; 27: 612–20
36. Ramallo-Fariña Y, García-Bello MA, García-Pérez L, et al. Effectiveness of internet-based multicomponent interventions for patients and health care professionals to improve clinical outcomes in type 2 diabetes evaluated through the INDICA study: multiarm cluster randomized controlled trial. JMIR Mhealth Uhealth 2020; 8: e18922.
37. Sadanshiv M, Jeyaseelan L, Kirupakaran H, Sonwani V, Sudarsanam TD. Feasibility of computer-generated telephonic message-based follow-up system among healthcare workers with diabetes: a randomized controlled trial. BMJ Open Diabetes Res Care 2020; 8: e001237
38. Whittemore R, Vilar-Compte M, De La Cerda S, et al. ¡Sí, Yo Puedo Vivir Sano con Diabetes! A self-management randomized controlled pilot trial for low-income adults with type 2 diabetes in Mexico City. Curr Dev Nutr 2020; 4: nzaa074
39. Xu R, Xing M, Javaherian K, Peters R, Ross W, Bernal-Mizrachi C. Improving HbA 1c with glucose self-monitoring in diabetic patients with EpxDiabetes, a phone call and text message-based telemedicine platform: a randomized controlled trial. Telemed J E Health 2020; 26: 784–93.
40. Omar MA, Hasan S, Palaian S, Mahameed S. The impact of a self-management educational program coordinated through WhatsApp on diabetes control. Pharm Pract (Granada) 2020; 18: 1841
41. Anzaldo-Campos MC, Contreras S, Vargas-Ojeda A, Menchaca-Díaz R, Fortmann A, Philis-Tsimikas A. Dulce wireless Tijuana: a randomized control trial evaluating the impact of project Dulce and short-term mobile technology on glycemic control in a family medicine clinic in Northern Mexico. Diabetes Technol Ther 2016; 18: 240–51
42. Gong E, Baptista S, Russell A, et al. My Diabetes Coach, a mobile app-based interactive conversational agent to support type 2 diabetes self-management: randomized effectiveness-implementation trial. J Med Internet Res 2020; 22: e20322
43. Kleinman NJ, Shah A, Shah S, Phatak S, Viswanathan V. Improved medication adherence and frequency of blood glucose self-testing using an m-Health platform versus usual care in a multisite randomized clinical trial among people with type 2 diabetes in India. Telemed J E Health 2017; 23: 733–40
44. Ku EJ, Park JI, Jeon HJ, Oh T, Choi HJ. Clinical efficacy and plausibility of a smartphone-based integrated online real-time diabetes care system via glucose and diet data management: a pilot study. Intern Med J 2020; 50: 1524–32
45. Kumar DS, Prakash B, Subhash Chandra BJ, et al. Technological innovations to improve health outcome in type 2 diabetes mellitus: a randomized controlled study. Clin Epidemiol Glob Health 2021; 9: 53–56
46. Li J, Wei D, Liu S, et al. Efficiency of an mHealth app and chest-wearable remote exercise monitoring intervention in patients with type 2 diabetes: a prospective, multicenter randomized controlled trial. JMIR Mhealth Uhealth 2021; 9: e23338
47. Yu Y, Yan Q, Li H, et al. Effects of mobile phone application combined with or without self-monitoring of blood glucose on glycemic control in patients with diabetes: a randomized controlled trial. J Diabetes Investig 2019; 10: 1365–71
48. Zhou W, Chen M, Yuan J, Sun Y. Welltang – a smart phone-based diabetes management application – improves blood glucose control in Chinese people with diabetes. Diabetes Res Clin Pract 2016; 116: 105–10.
49. Zhou W, Chen M, Yuan J, Sun Y. Welltang – a smart phone-based diabetes management application – improves blood glucose control in Chinese people with diabetes. Diabetes Res Clin Pract 2016; 116: 105–10.
50. McLeod M, Stanley J, Signal V, et al. Impact of a comprehensive digital health programme on HbA 1c and weight after 12 months for people with diabetes and prediabetes: a randomised controlled trial. Diabetologia 2020; 63: 2559–70
51. Ralston JD, Hirsch IB, Hoath J, Mullen M, Cheadle A, Goldberg HI. Web-based collaborative care for type 2 diabetes: a pilot randomized trial. Diabetes Care 2009; 32: 234–39
52. Ramadas A, Chan CKY, Oldenburg B, Hussein Z, Quek KF. Randomised-controlled trial of a web-based dietary intervention for patients with type 2 diabetes: changes in health cognitions and glycemic control. BMC Public Health 2018; 18: 716
53. Tang PC, Overhage JM, Chan AS, et al. Online disease management of diabetes: engaging and motivating patients online with enhanced resources-diabetes (EMPOWER-D), a randomized controlled trial. J Am Med Inform Assoc 2013; 20: 526–34
54. Zhou P, Xu L, Liu X, Huang J, Xu W, Chen W. Web-based telemedicine for management of type 2 diabetes through glucose uploads: a randomized controlled trial. Int J Clin Exp Pathol 2014; 7: 8848–54.

**Ineligible study design**

1. Clemins EL et al. (2018). “Improving blood pressure control using smart technology.” Telemed J E Health 24(4): 222.
2. Hsu Wc, L. et al. (2016). "Utilization of a Cloud-Based Diabetes Management Program for Insulin Initiation and Titration Enables Collaborative Decision Making Between Healthcare Providers and Patients." 18(2): 59.
3. Lopez-Torres J, R. et al. (2015). "Effectiveness of a telemedicine programme for patients with metabolic syndrome." 23(2): 161.
4. Park MJ et al. (2009). “Cellular phone and Internet-based individual intervention on blood pressure and obesity in obese patients with hypertension.” Int J Med Inform 78(10): 704.
5. Pichayapinyo, P., et al. (2019). "Feasibility study of automated interactive voice response telephone calls with community health nurse follow‐up to improve glycaemic control in patients with type 2 diabetes." International Journal of Nursing Practice (John Wiley & Sons, Inc.) 25(6): N.PAG-N.PAG.
6. Powers, B. J., et al. (2009). "The effect of a hypertension self-management intervention on diabetes and cholesterol control." American Journal of Medicine 122(7): 639-646.
7. Waki K et al. (2015). “DialBetics With a Multimedia Food Recording Tool, FoodLog: Smartphone- Based Self-Management for Type 2 Diabetes.” J Diabetes Sci Technol 9(3): 534.

**Ineligible intervention**

1. Bergenstal, R. M., et al. (2019). "Automated insulin dosing guidance to optimise insulin management in patients with type 2 diabetes: a multicentre, randomised controlled trial." Lancet 393 North American Edition (10176): 1138-1148.
2. Bradway, M., et al. (2018). "Analysing mHealth usage logs in RCTs: Explaining participants' interactions with type 2 diabetes self-management tools." PloS one 13(8): e0203202.
3. Debon R et al. (2020). “Effects of using a mobile health application on the health conditions of patients with arterial hypertension: A pilot trial in the context of Brazil's Family Health Strategy.” Sci Rep 10(1): 6009.
4. de Vasconcelos Hca, L. et al. (2018). "Telecoaching programme for type 2 diabetes control: a randomised clinical trial." 27(19): 1115.
5. Eborall, H. C., et al. (2015). "Explaining engagement in self-monitoring among participants of the DESMOND Self-monitoring Trial: a qualitative interview study." Family Practice 32(5): 596-602.
6. Fortmann A.I. et al. (2016). "Diabetes distress affects responsiveness to an mhealth self-management intervention among hispanics with type 2 diabetes (dulce digital)." 65: A202.
7. Georgsson, M. and N. Staggers (2016). "An evaluation of patients' experienced usability of a diabetes mHealth system using a multi-method approach." Journal of Biomedical Informatics 59: 115-129.
8. Glaser E,et al. (2017). "The impact of a patient web communication intervention on reaching treatment suggested guidelines for chronic diseases: a randomized controlled trial." Date of Publication: December 20.
9. Grey Eb, et al. (2019). "Effects of a Web-Based, Evolutionary Mismatch-Framed Intervention Targeting Physical Activity and Diet: a Randomised Controlled Trial." 26(6): 645.
10. Huang, M. C., et al. (2017). "The effectiveness of multimedia education for patients with type 2 diabetes mellitus." Journal of Advanced Nursing (John Wiley & Sons, Inc.) 73(4): 943-954. Ipjian Ml, J. C. S. (2017). "Smartphone technology facilitates dietary change in healthy adults." 33: 343.
11. Kempf K, et al. (2017). "Efficacy of the Telemedical Lifestyle intervention Program TeLiPro in Advanced Stages of Type 2 Diabetes: a Randomized Controlled Trial." 40(7): 863.
12. Leichter S et al. (2013). "Impact of remote management of diabetes via computer: the 360 study--a proof-of-concept randomized trial." 15(5): 434.
13. Liu S, et al. (2018). "Effectiveness of User- and Expert-Driven Web-based Hypertension Programs: an RCT." 54(4): 576.
14. Nicolucci A, et al. (2015). "A Randomized Trial on Home Telemonitoring for the Management of Metabolic and Cardiovascular Risk in Patients with Type 2 Diabetes." 17(8): 563.
15. Prabhakaran D, et al. (2019). "Effectiveness of an mHealth-Based Electronic Decision Support System for Integrated Management of Chronic Conditions in Primary Care: the mWellcare Cluster-Randomized Controlled Trial." 139(3): 380.
16. Prabhakaran D, et al. (2018). "Effectiveness of mhealth based decision support system for integrated management of chronic conditions in primary care: the mwellcare trial." 138(25): e758.
17. Schoenthaler A, et al. (2020). "Development and Evaluation of a Tailored Mobile Health Intervention to Improve Medication Adherence in Black Patients With Uncontrolled Hypertension and Type 2 Diabetes: pilot Randomized Feasibility Trial." 8(9): e17135.

Table 3 Characteristics of the included studies

| Reference; country | Sample size at baseline | Mean Age (years)  Mean ±SD or n (%) | Gender  Female n (%) | Study design; duration | mHealth type |
| --- | --- | --- | --- | --- | --- |
| Bobrow et al.,  2016 [7];  South Africa | CG: 457  IG1: 457  IG2: 458 | CG: 54.7±11.6  IG1: 53.9±11.2  IG2: 54.2± 11.6 | CG: 331 (72%) IG1: 331 (72%)  IG2: 331 (72%) | Single-blind RCT;  12 months | SMS |
| Jahan et al., 2020 [8]; Bangladesh | CG: 211  IG: 209 | CG: 47.8±8.6  IG: 46.4±8.3 | CG: 177 (84%) IG: 184 (88%) | Prospective single-centre RCT;  5 months | SMS |
| Rehman et al.,  2019 [26]; Pakistan | CG: 60  IG: 60 | Not reported in a format required | Not reported in a format required | Prospective RCT;  3 months | SMS |
| Gong et al., 2020 [9]; China | CG: 240  IG: 240 | CG: 59.3±7.4  IG: 58.2±7.5 | CG: 103 (47%) IG: 99 (44%) | Multicenter RCT;  6 months | Smartphone app |
| Li et al., 2019 [10]; China | CG: 276  IG: 186 | CG: 61.3±6.4  IG: 61.7±6.3 | CG: 182 (66%) IG: 111 (60%) | Prospective cluster RCT;  6 months | Smartphone app |
| Sun et al., 2020 [18]; China | CG: 60  IG: 60 | CG: 53.4±8.8  IG: 52.4±9.5 | CG: 30 (52%)  IG: 28 (48%) | RCT,  3 months | Smartphone app |
| Wang et al., 2020 [19]; China | CG: 87  IG: 87 | 35-59 (35, 47%)  60-86 (40, 53%) | CG: 25 (33%)  IG: 26 (34%) | Multicenter RCT;  6 months | SMS |
| Wan et al., 2018 [20]; China | CG: 87  IG: 87 | 35-59 (36, 46%)  60-86 (42, 54%) | CG 26 (33%)  IG 29 (36%) | Multicenter RCT;  3 months | SMS |
| Bhandari et al., 2022 [11]; Nepal | CG: 100  IG: 100 | CG: 51.7±9.2  IG: 49.2±9.8 | CG: 47 (47%)  IG: 42 (42%) | Unblinded RCT;  3 months | SMS |
| Kingue et al.,  2013 [27]; Cameroon | CG: 103  IG: 165 | CG: 57.6±12.1  IG: 59.9±10.4 | CG: 56 (55%)  IG: 105 (64%) | Prospective RCT;  6 months | Mobile phone call |
| Prabhakaran et al., 2019 [28]; India | CG: 1856  IG: 1842 | CG: 54.5±10.9  IG: 55.8±11.0 | CG: 871 (47%) IG: 786 (43%) | Prospective multicenter RCT;  12 months | Smartphone app |
| Piette et al., 2012 [29]; Honduras and Mexico | CG: 101  IG: 99 | CG: 57.1±1.1  IG: 58.0±1.3 | CG: 62 (68%)  IG: 59 (66%) | RCT;  1.5 months | Mobile phone call |
| Pan et al., 2018 [21]; China | CG: 55  IG: 55 | CG: 57.8±10.9  IG: 56.6±9.8 | CG: 31 (56%)  IG: 26 (50%) | RCT;  6 months | Smartphone app |
| Zhai et al., 2020 [22]; China | CG: 192  IG: 192 | CG: 69.4±9.7  IG: 68.5±7.9 | CG: 141 (73%) IG: 124 (65%) | Cluster RCT;  3 months | SMS |
| Yuting et al., 2023 [23]; China | CG: 68  IG: 66 | CG: 62.1±10.7  IG: 61.4±11.7 | CG: 30 (44%)  IG: 21 (32%) | RCT;  3 months | Smartphone app |
| He et al., 2017 [30]; Argentina | CG: 689  IG: 743 | CG: 55.5±13.0  IG: 56.1±13.6 | CG: 378 (53%) IG: 394 (53%) | Cluster RCT;  18 months | SMS |
| Maslakpak et al., 2016 [24]; Iran | CG: 41  IG1: 41  IG2: 41 | CG: 50.5±8.14  IG1: 53.7±6.94  IG2: 50.3±10.5 | CG: 29 (71%)  IG1: 27 (66%) IG2: 31 (76%) | RCT;  3 months | SMS |
| Bozorgi et al., 2021 [25]; Iran | CG: 60  IG: 60 | CG: 51.6±9.4  IG: 52.0±8.1 | CG: 24 (40%)  IG: 25 (42%) | RCT;  6 months | Smartphone app |
| Zhang et al., 2022 [32]; China | CG: 143  IG: 164 | CG: 62.6±10.1  IG: 56.7±9.3 | CG: 57 (64.8%)  IG: 60 (57.7%) | RCT;  6 months | Smartphone app |
| David et al., 2023 [33]; Brazil | CG: 45  IG: 186 | CG: 55.0±8.7  IG: 55.5±9.7 | CG: 19 (42.2%)  IG: 92 (49.5%) | RCT;  6 months | SMS |
| Kes et al., 2021 [34]; Turkey | CG: 46;  IG: 46 | CG: 52.2±6.2  IG: 54.9±6.6 | CG: 21 (55.3%)  IG: 20 (51.3%) | Single-blind RCT;  3 months | SMS |
| Zhou et al., 2022 [35]; China | CG: 1933 IG: 3790 | CG: 61.6±9.4  IG: 61.6±9.4 | CG: 610 (53.8%)  IG: 1655 (55.4%) | Cluster RCT;  12 months | SMS |
| Ionov et al., 2020[36]; Russia | CG: 82  IG: 164 | Not reported in a format required | CG: 65 (40.6%) IG: 31 (38.8%) | Prospective RCT;  3 months | Smartphone app |

RCT: randomised control trial; IG: intervention group; CG: control group; SMS: short message service; BP: blood pressure


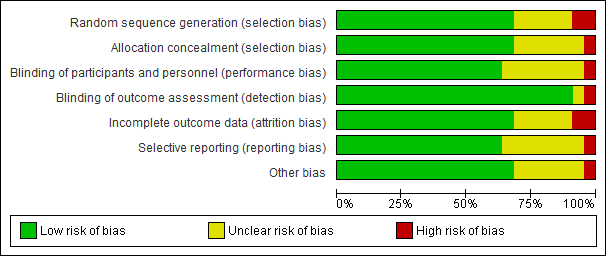


Figure 1 Risk of bias graph of the included studies


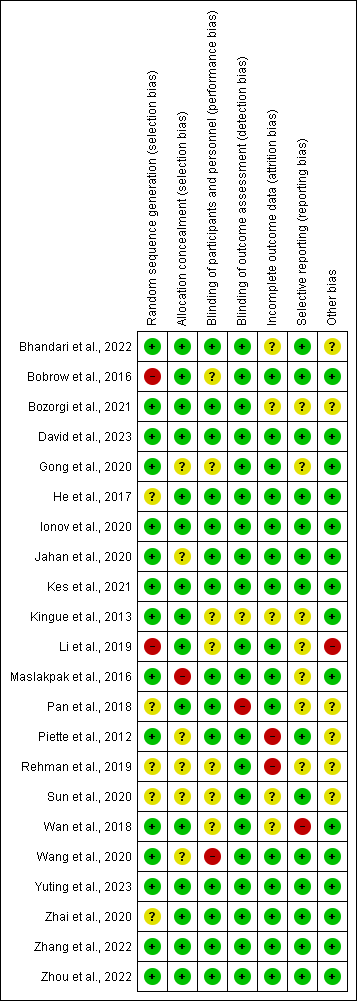


Figure 2 Risk of bias summary of the included studies


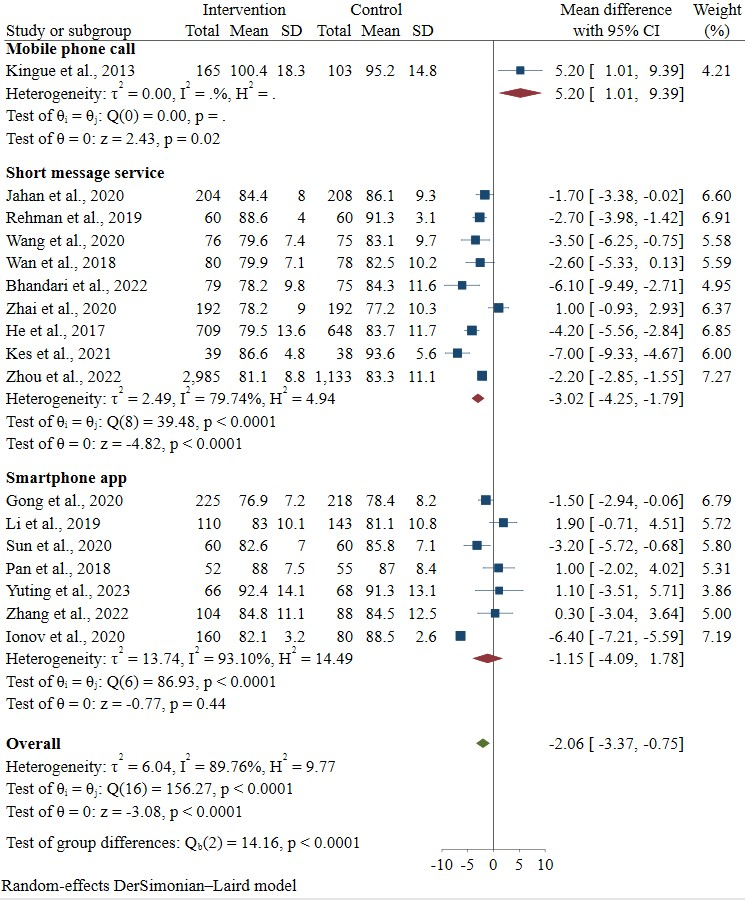
Figure 3 Forest plot of the mean difference in DBP between the intervention and control groups


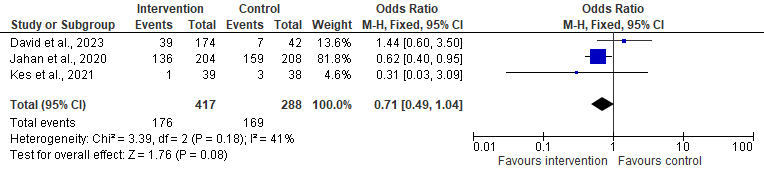


Figure 4 Meta-analysis of dichotomous outcome measurements for low-salt diets


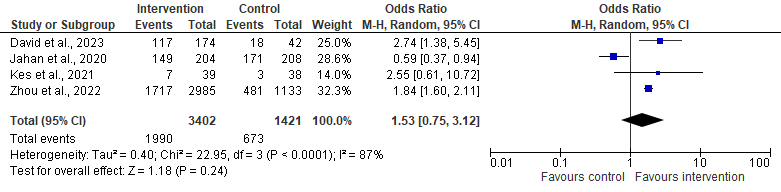


Figure 5 Meta-analysis of dichotomous outcome measurements for PA


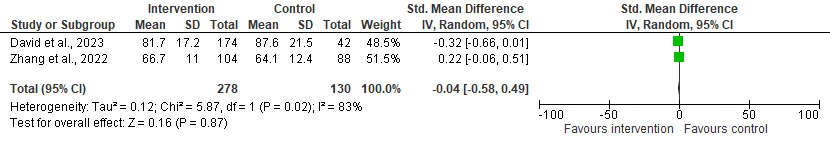


Figure 6 Meta-analysis of continuous outcome measurements for weight loss


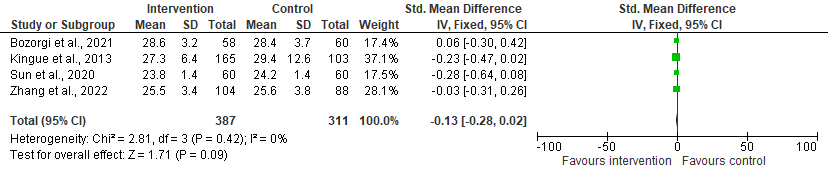


Figure 7 Meta-analysis of dichotomous outcome measurements for BMI


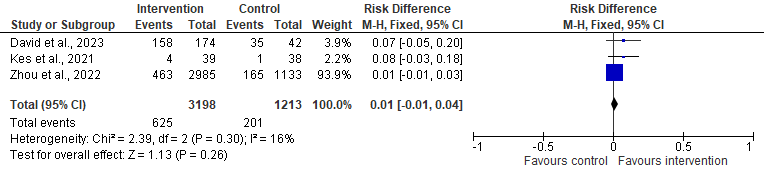


Figure 8 Meta-analysis of dichotomous outcome measurements for alcohol reduction

Table 4 Subgroup analyses of mean differences in SBP and DBP

| Subgroup analyses | No. of  Studies | Net  Change,  mm Hg | 95% CI | Heterogeneity across the studies | | Heterogeneity between groups  (p-value) |
| --- | --- | --- | --- | --- | --- | --- |
|  |  |  |  | % I^2^ | p-value |  |
| **SBP** |  |  |  |  |  |  |
| Total | 19 | -4.43 | -6.19 to -2.67 | 92.06 | <0.0001 | <0.0001 |
| Income-economy |  |  |  |  |  | 0.22 |
| Lower middle income | 5 | -2.61 | -5.99 to 0.77 | 88.71 | <0.0001 |  |
| Upper middle income | 14 | -5.01 | -6.83 to -3.20 | 87.30 | <0.0001 |  |
| Intervention duration |  |  |  |  |  | 0.95 |
| < 12 months | 16 | -4.41 | -6.27 to -2.55 | 88.17 | <0.0001 |  |
| ≥ 12 months | 3 | -4.55 | -8.29 to -0.81 | 93.73 | <0.0001 |  |
| Publication year |  |  |  |  |  | <0.0001 |
| > 2019 | 11 | -6.79 | -8.68 to -4.91 | 81.96 | <0.0001 |  |
| ≤ 2019 | 8 | -1.91 | -3.68 to -0.15 | 81.20 | <0.0001 |  |
| Age group |  |  |  |  |  | 0.08 |
| < 54 years | 4 | -8.07 | -12.85 to -3.30 | 85.77 | <0.0001 |  |
| 54-60 years | 10 | -2.55 | -4.10 to -1.00 | 78.67 | <0.0001 |  |
| > 60 years | 4 | -4.74 | -9.00 to -0.48 | 88.15 | <0.0001 |  |
| Gender |  |  |  |  |  | 0.24 |
| Female | 12 | -3.71 | -6.16 to -1.26 | 94.50 | <0.0001 |  |
| Male | 6 | -5.65 | -7.78 to -3.51 | 60.69 | 0.03 |  |
| Country |  |  |  |  |  | 0.93 |
| China | 10 | -4.52 | -6.77 to -2.26 | 86.69 | <0.0001 |  |
| Other countries | 9 | -4.36 | -6.83 to -1.90 | 91.53 | <0.0001 |  |
| **DBP** |  |  |  |  |  |  |
| Total | 17 | -2.06 | -3.37 to -0.75 | 89.76 | <0.0001 | 0.02 |
| Income-economy |  |  |  |  |  | 0.77 |
| Lower middle income | 4 | -1.69 | -4.43 to 1.06 | 83.31 | <0.0001 |  |
| Upper middle income | 13 | -2.15 | -3.70 to -0.60 | 91.11 | <0.0001 |  |
| Intervention duration |  |  |  |  |  | 0.32 |
| < 12 months | 15 | -1.80 | -3.50 to -0.11 | 90.35 | <0.0001 |  |
| ≥ 12 months | 2 | -3.11 | -5.06 to -1.16 | 85.30 | 0.01 |  |
| Publication year |  |  |  |  |  | 0.15 |
| > 2019 | 11 | -2.77 | -4.45 to -1.09 | 91.40 | <0.0001 |  |
| ≤ 2019 | 6 | -0.66 | -2.99 to 1.67 | 85.61 | <0.0001 |  |
| Age group |  |  |  |  |  | 0.05 |
| < 54 years | 4 | -4.37 | -7.06 to -1.67 | 79.04 | <0.0001 |  |
| 54-60 years | 7 | -1.18 | -3.12 to 0.77 | 80.35 | 0.02 |  |
| > 60 years | 4 | 0.20 | -2.31 to 2.71 | 83.73 | <0.0001 |  |
| Gender |  |  |  |  |  | 0.16 |
| Female | 10 | -1.23 | -2.82 to 0.35 | 85.90 | <0.0001 |  |
| Male | 6 | -3.38 | -5.89 to -0.87 | 89.00 | <0.0001 |  |
| Country |  |  |  |  |  | 0.03 |
| China | 10 | -0.99 | -2.14 to 0.15 | 65.90 | 0.02 |  |
| Other countries | 7 | -3.62 | -5.67 to -1.57 | 90.76 | <0.0001 |  |


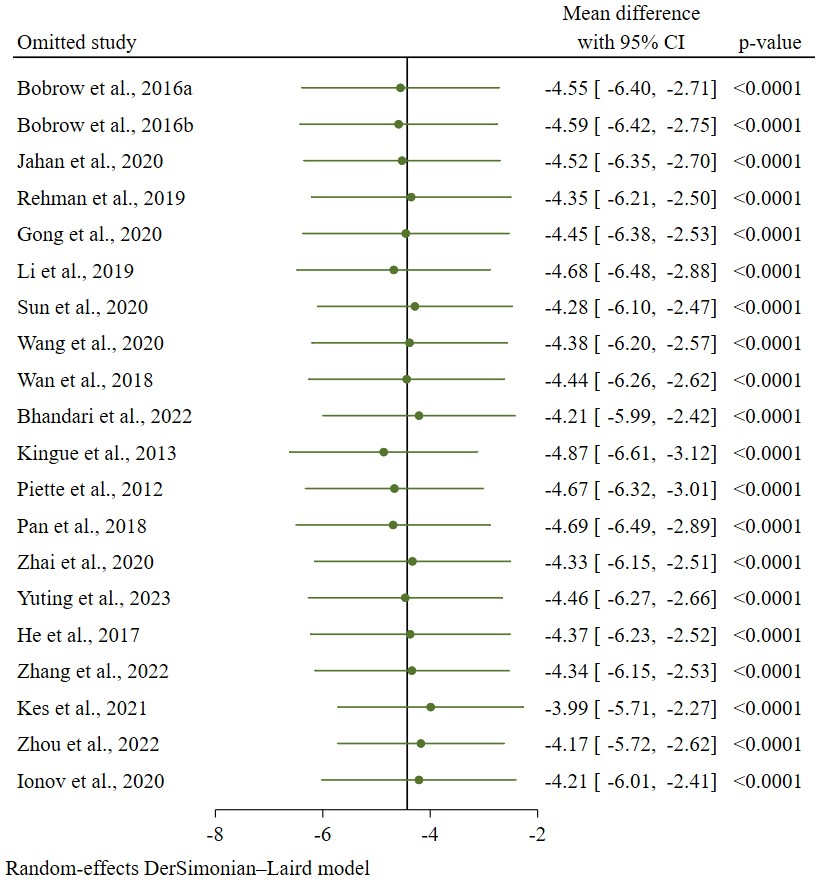


Figure 4 Sensitivity analyses for the SBP outcome


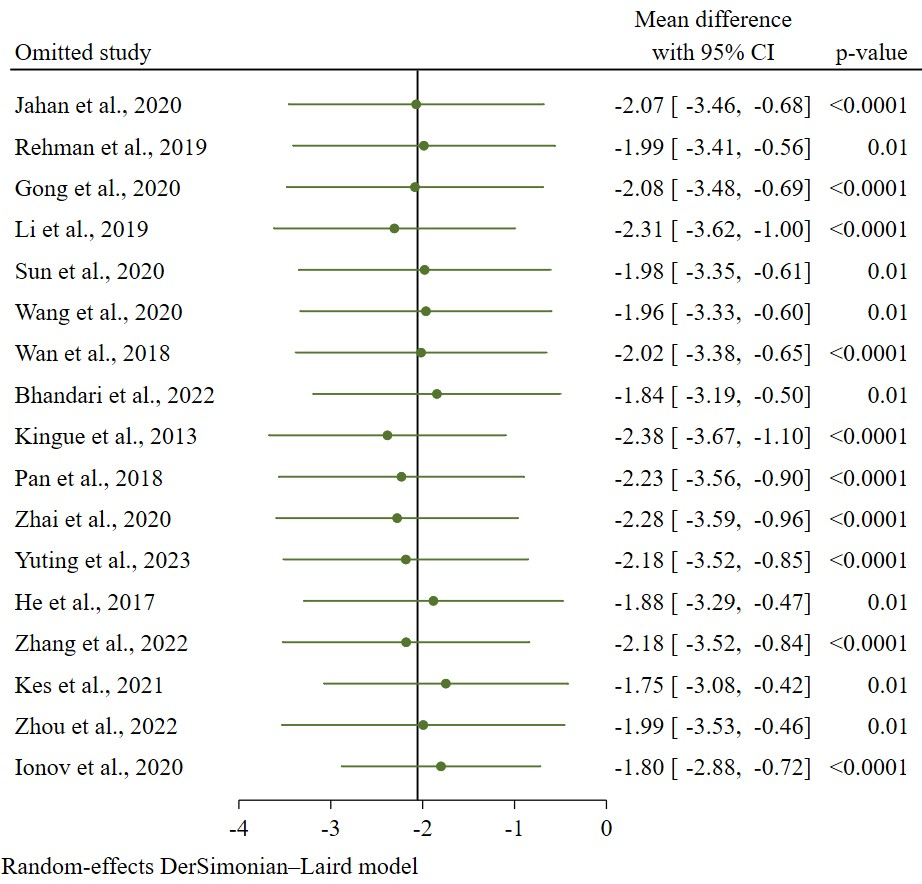


Figure 5 Sensitivity analyses for DBP


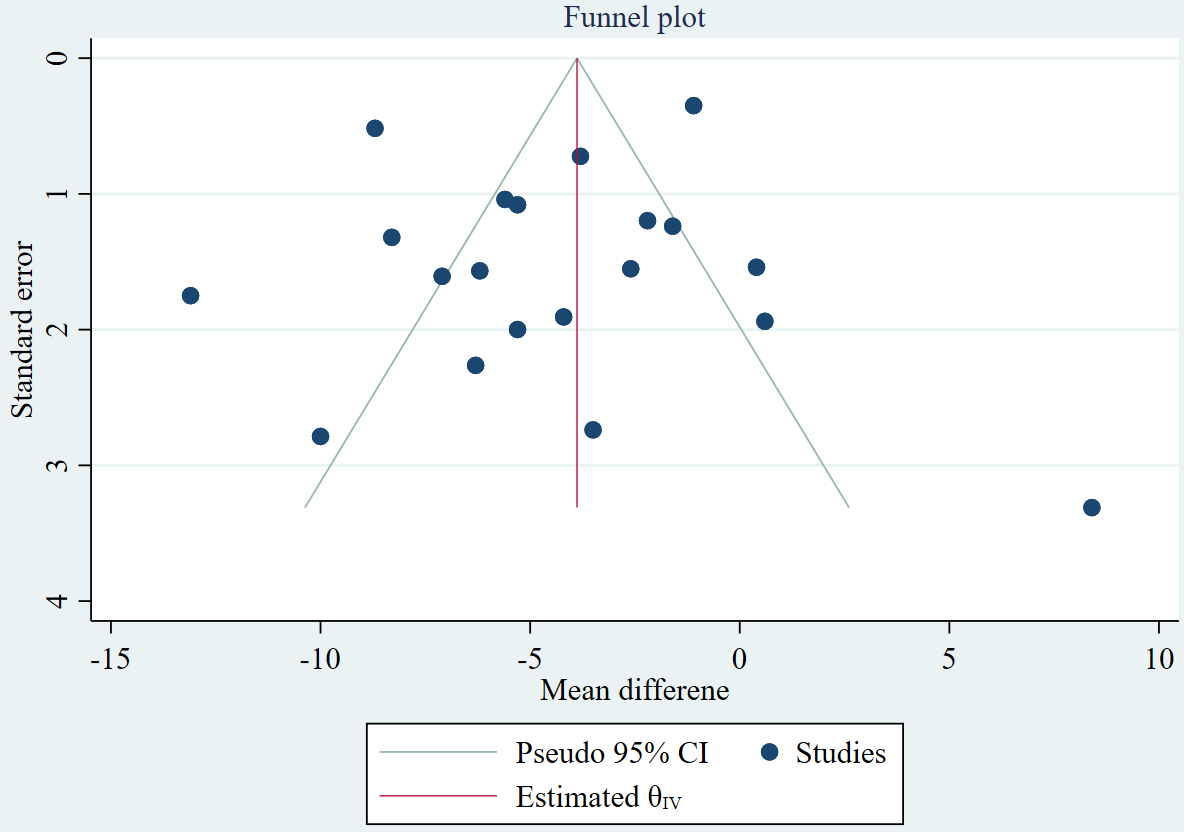


Figure 6 Funnel plot for risk of publication bias for the SBP outcome


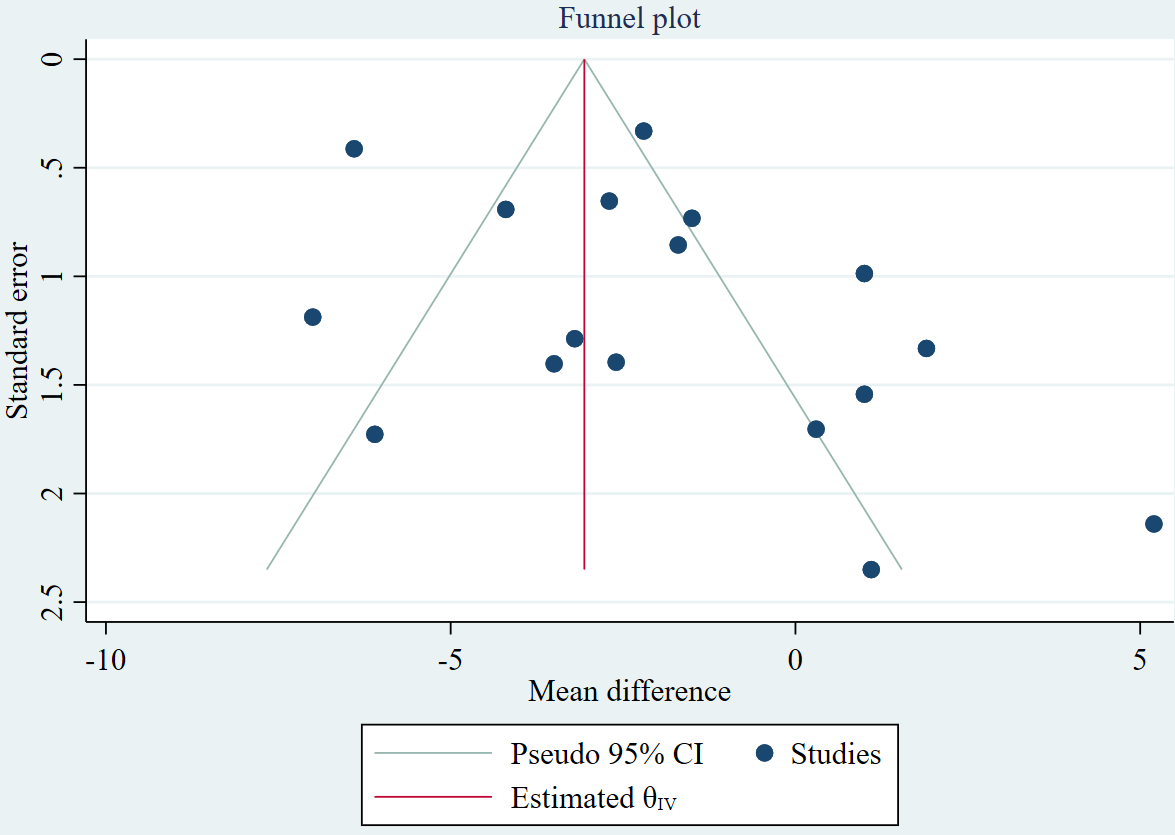


Figure 7 Funnel plot for risk of publication bias for the DBP outcome
